# Challenges, Power-Device Progress, and Emerging Harsh-Environment Applications for Ultrawide-Bandgap Diamond Semiconductors

**DOI:** 10.3390/ma19163529

**Published:** 2026-08-20

**Authors:** Nuwayyir Alshammari, Mulpuri V. Rao, Qiliang Li

**Affiliations:** 1 Department of Electrical Engineering, George Mason University, Fairfax, VA 22030, USA; nalsham5@gmu.edu; 2 Department of Advanced Manufacturing and Robotics, Peking University, Beijing 100871, China

**Keywords:** diamond semiconductor, diamond electronics, ultrawide-bandgap semiconductors, diamond power devices, hydrogen-terminated diamond, diamond MOSFETs, diamond Schottky diodes, diamond p–i–n diodes, diamond memory devices

## Abstract

Diamond has emerged as a promising ultrawide-bandgap semiconductor material for next-generation electronics because of its unique combination of a wide bandgap, high critical electric field, superior carrier transport properties, exceptionally high thermal conductivity, and strong chemical and radiation stability. Over the past two decades, progress in crystal growth, substrate engineering, surface control, dielectric integration, and device fabrication has advanced diamond electronics beyond early proof-of-concept demonstrations. The review connects material properties, growth, doping, defects, and figures of merit with reported performance in hydrogen-terminated field-effect transistors, MOSFETs, Schottky and p–i–n diodes, and related power-device architectures. Emerging opportunities in ultraviolet photodetectors, multifunctional electronics, and memory-oriented diamond devices are also briefly considered. Among the device classes reviewed, diamond diodes currently show the strongest evidence of high-voltage capability, whereas transistor development remains constrained by threshold-voltage control, normally off operation, contact resistance, interface stability, and reliability. Diamond is therefore more likely to complement than replace established SiC and GaN technologies, particularly in specialized high-field, high-temperature, radiation-rich, and chemically demanding applications. Broader deployment will require scalable low-defect wafers, reliable n-type doping, stable interfaces and contacts, and more cost-effective manufacturing.

## 1. Introduction

Since the mid-1950s, silicon (Si) has been the dominant material in electronics and, in particular, the semiconductor industry. Silicon-based devices, however, have limitations in terms of heat dissipation, efficiency, voltage blocking capability, and maximum junction temperature [1,2]. In recent years, wide-bandgap (WBG) semiconductor materials such as silicon carbide (SiC) and gallium nitride (GaN) have enabled new generations of semiconductor devices. These WBG materials generally offer higher breakdown voltage, higher saturation velocity, and improved thermal performance compared with Si [3,4,5]. Furthermore, WBG materials can sustain high voltages and currents with reduced conduction losses and can operate at higher frequencies, making them attractive for high-power and high-frequency electronic applications. Among the available WBG materials, GaN-based devices are particularly important for high-frequency operation. The high electron mobility of GaN transistors enables efficient high-frequency switching, making them useful for fast switching, RF electronics, and power-conversion applications. Their radiation tolerance has also motivated interest in radiation-environment applications.

Table 1 summarizes representative room-temperature electrical and thermal properties of Si, 4H-SiC, and GaN that are commonly used to compare conventional and wide-bandgap power-semiconductor platforms. SiC power MOSFETs and Schottky diodes already compete directly with their Si counterparts in many applications [3,6,7,8]. GaN technology has also developed rapidly from its established role in LED production toward high-frequency and high-voltage electronics, particularly GaN-based high-electron-mobility transistors (HEMTs) [7,9,10]. Despite these advantages, WBG technologies remain constrained by trade-offs between material quality, cost, and device reliability, and many reported devices still require continued improvements in long-term stability and manufacturing scalability. Similar or greater challenges are present in emerging ultrawide-bandgap platforms.

Ultrawide-bandgap (UWBG) semiconductors are commonly defined as materials with bandgaps wider than those of GaN (∼3.4 eV) and SiC (∼3.2–3.3 eV). These materials have become the subject of intensive research and development because of their higher breakdown fields, which generally increase with bandgap. They are also being explored for use in harsh environments, due to their high thermal stability and radiation resistance across a broad range of materials and devices [11]. However, many UWBG technologies are still in early stages of development, where material quality, doping control, and scalable device fabrication continue to present significant challenges. Table 2 provides an overview of selected material properties for several WBG and UWBG semiconductors [12].

Table 2 emphasizes broadly comparable material properties. The effects of donor and acceptor densities, as well as surface and interface charge, are discussed later in device-specific contexts because they depend strongly on dopant activation, compensation, surface termination, and device architecture.

Among the UWBG materials listed in Table 2, diamond offers a distinctive combination of properties compared to Si and other WBG or UWBG semiconductors [3]. In addition to being the hardest known bulk material in nature, diamond exhibits outstanding intrinsic electronic properties, including a wide bandgap of 5.47 eV, a theoretical breakdown field exceeding 10 MV cm^−1^, high intrinsic electron and hole mobilities exceeding 2000 cm^2^ V^−1^ s^−1^, and saturated drift velocities as high as 2.3 × 10^7^ cm s^−1^ for electrons and 1.4 × 10^7^ cm s^−1^ for holes [13,14,15,16,17,18]. Its thermal conductivity is among the highest reported for bulk materials, which is critical for efficient heat dissipation in device operation [12]. However, practical device performance is often limited by incomplete dopant ionization, compensation effects, interface-state density, and substrate defect density, which prevent present devices from reaching their intrinsic material limits.

Taken together, the large bandgap, high thermal conductivity, high carrier mobilities, and high saturation velocities summarized in Table 2 make diamond well-suited for UWBG applications. These properties suggest that diamond is particularly attractive for high-power and high-frequency electronics, vacuum switching structures, electron-emission devices, radiation detectors, traveling-wave tube components, and thermionic energy-conversion systems [12]. This review focuses on how these material characteristics influence the design, performance, and practical limitations of diamond-based power devices, with particular attention to metal–oxide–semiconductor field-effect transistors (MOSFETs) and related device structures.

**Figure 1:**
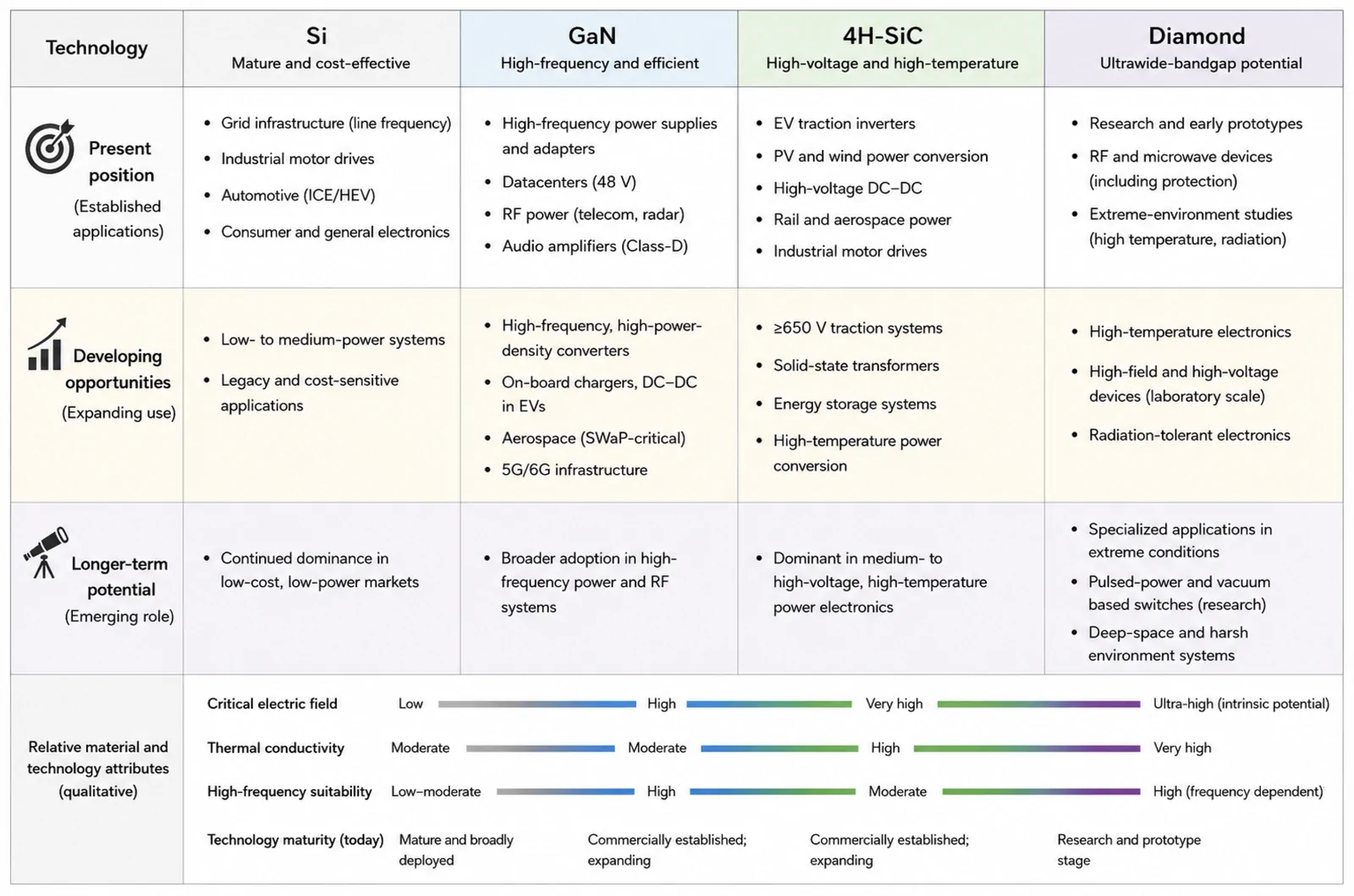
Qualitative roadmap comparing the present position, developing opportunities, longer-term potential, and relative attributes of Si, GaN, 4H-SiC, and diamond for power-electronics applications. The application domains and technology attributes are qualitative, may overlap, and are expected to evolve with advances in material quality, device reliability, and manufacturing scalability.

Diamond should not be viewed as a direct replacement for Si, GaN, or 4H-SiC across all power-electronics applications. Instead, its most promising role lies in specialized high-field, high-temperature, and harsh-environment systems where its intrinsic properties may justify the present limitations in cost, wafer size, and manufacturing maturity. Figure 1 provides a qualitative roadmap comparing the present position and prospective application domains of these semiconductor technologies.

Recent work on diamond materials and devices spans growth, doping, surface physics, and power-device demonstrations, but these topics often appear in isolation. Previous reviews have provided valuable coverage of diamond material development, surface-channel electronics, and specific device classes [19,20,21]. Building on the literature, the present review connects material-level constraints, including substrate quality, doping, defects, and interface control, with reported device-level performance and practical power-electronics requirements. It further considers emerging memory, MEMSs/NEMSs, and harsh-environment applications that illustrate broader device opportunities enabled by diamond’s material properties.

This review is structured as follows: Section 2 summarizes the electronic properties of diamond that are most relevant for electronic devices. Section 3 addresses its physical properties, with emphasis on temperature behavior and breakdown. Section 4 discusses optoelectronic properties, particularly UV photodetection. Section 5 revisits common figures of merit (FOMs) for power devices in the context of diamond. Section 6 surveys recent progress in diamond electronic devices, including diodes, FETs, memory-related concepts, and related structures. While substantial progress has been made, diamond electronics remain in a transitional stage between laboratory-scale demonstrations and scalable power-device platforms. Accordingly, understanding the interplay between material growth, defect control, doping physics, and device architecture is essential to evaluating both the opportunities and the remaining barriers to practical diamond power devices.

## 2. Electronic Properties of Diamond

### 2.1. Substrate and Growth

Although polycrystalline diamond shares many of the intrinsic properties of natural diamond, grain boundaries and impurity incorporation, for example, nitrogen or silicon, can strongly degrade carrier transport, increase carrier scattering, and introduce leakage paths that reduce breakdown performance. For applications that demand high carrier mobility and efficient charge collection, single-crystalline diamond (SCD) is therefore preferred. In recent years, substantial progress has been made in homoepitaxial diamond growth, and several groups have demonstrated high-quality deposition on commercially available SCD substrates [19,22,23,24,25]. These substrates are typically synthesized either by chemical vapor deposition (CVD) or by high-pressure, high-temperature (HPHT) techniques [19]. CVD growth enables homoepitaxial diamond layers on existing seeds with controlled doping and thickness, whereas HPHT growth is primarily used to produce bulk Type-IIa crystals that serve as starting substrates.

Both synthetic and natural diamond contain some nitrogen (N) impurities and are commonly classified according to their impurity content [26]. Type-I diamond exhibits nitrogen concentrations that are detectable by infrared (IR) spectroscopy. The nitrogen atoms can substitute for carbon and either form aggregated complexes, Type Ia, or remain isolated, Type Ib. Type-Ia material is further subdivided into Type IaA, in which nitrogen atoms occur in pairs, and Type IaB, in which four nitrogen atoms surround a vacancy in a roughly tetrahedral arrangement. Type-II diamond, in contrast, shows nitrogen levels below the IR detection limit and often contains other dopants such as boron. Within this class, Type-IIa crystals have the lowest overall impurity concentration and are regarded as the purest form of diamond, whereas Type-IIb crystals contain sufficient boron to exhibit p-type semiconducting behavior [19].

For diamond electronic devices, two substrate factors are especially important: defect density and available substrate area [19]. HPHT growth usually gives Type-IIa substrates with low dislocation density, often below about $10^{3}$ cm^−2^. The main limitation of HPHT diamond remains the limited substrate size. In most cases, HPHT substrates are still limited to about 1 cm^2^ or less. CVD growth offers a more scalable route to larger-area substrates. Reported CVD wafer sizes have reached about 3.5 inches in diameter [27,28]. The trade-off is that CVD diamond often has higher defect density, commonly around 10^7^–10^9^ cm^−2^ [29,30]. This balance between size and crystal quality is still a major issue for large-area, high-voltage diamond devices. These devices need both low defect density and wafer-scale processing. Dislocations that begin in the substrate may also continue into the epitaxial layer, where they can create leakage paths or lead to early breakdown. The influence of defect density becomes increasingly important as the device area increases. As a simple geometrical estimate, an active area of $100\times 100\;\mu m^{2}$ corresponds to $10^{-4}\;{\mathrm{cm}}^{2}$. Multiplying this area by the reported areal defect densities gives an expected value of approximately 0.1 defect intersections at $10^{3}\;{\mathrm{cm}}^{-2}$, compared with approximately $10^{3}$–$10^{5}$ intersections at $10^{7}$–$10^{9}\;{\mathrm{cm}}^{-2}$ [29,30]. This estimate assumes a spatially uniform defect distribution and does not imply that every defect is electrically active. Nevertheless, because some defects can enhance the local electric field or form leakage paths [31,32,33,34], increasing the active area raises the probability of encountering performance-limiting defects and can therefore reduce device uniformity and fabrication yield.

Different methods have been explored to reduce these substrate limits. Mosaic CVD growth is one example. In this method, separate diamond tiles are joined to form a larger substrate. The effective area can exceed 5 cm^2^, but the joined regions can introduce strain, defects, or non-uniform electronic behavior. The bonding interfaces are often strained and defective, and device performance may be affected if the active region crosses these boundaries [35]. Heteroepitaxial growth is another option. Here, diamond is grown on a non-diamond substrate, such as iridium (Ir) or a related multilayer stack. Substrates larger than 3 inches have been demonstrated, making this approach attractive for wafer-scale growth. Still, the material often contains high dislocation densities, around 10^7^–10^9^ cm^−2^, together with more complex defect structures [36,37,38,39]. Since diamond has no lattice-matched substrate other than diamond itself, heteroepitaxial growth usually needs buffer layers, such as Ir/YSZ/Si stacks, to handle the large lattice mismatch.

Diamond substrates have improved through HPHT, CVD, mosaic growth, and heteroepitaxial growth. Even so, producing device-ready diamond wafers is still difficult. For power-device applications, the ideal substrate must combine large area, low defect density, uniform doping, and process compatibility. At present, achieving all of these at the same time remains challenging. Cost, growth rate, and wafer uniformity also need to improve before diamond power devices can move toward scalable manufacturing. A direct cost comparison between diamond, SiC, and GaN remains difficult because diamond devices have not yet reached standardized high-volume manufacturing. Nevertheless, the much smaller substrate size, slower growth, specialized processing, and uncertain fabrication yield place diamond at a clear economic disadvantage. Its near-term commercial potential is therefore more likely in specialized, high-value applications where extreme operating capability can justify the added cost.

### 2.2. Crystal Structure

**Figure 2:**
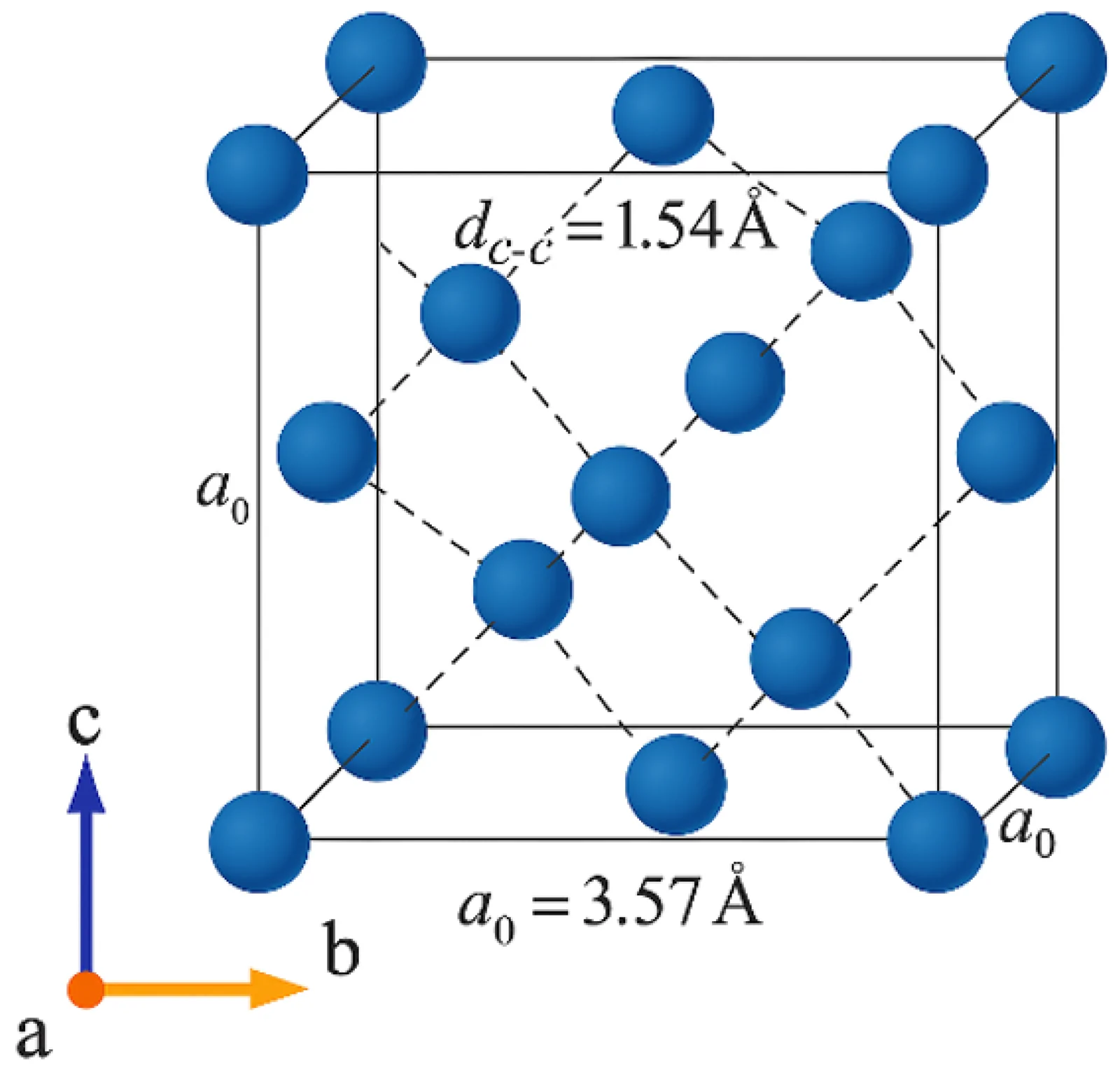
Diamond cubic crystal structure showing the tetrahedral bonding configuration and C–C bond length. Adapted from [40].

Diamond is a crystalline form of carbon with a face-centered cubic (FCC) structure. This structure is commonly referred to as the diamond cubic lattice. Each carbon atom is $sp^{3}$-hybridized and covalently bonded to four nearest neighbors in a tetrahedral configuration, forming a highly rigid three-dimensional covalent network. The C–C bond length is about 1.54 Å, and the lattice constant is 3.57 Å [40]. Structurally, the lattice can be viewed as two interpenetrating FCC sublattices displaced by one-quarter of the lattice constant along each crystallographic axis, forming the three-dimensional tetrahedral network illustrated in Figure 2 [40].

An important feature of diamond for device engineering is its anisotropy. Although it is often treated as isotropic in simplified models, several practical properties, including growth rate, impurity incorporation, surface chemistry, and carrier transport behavior, depend strongly on crystallographic orientation. This anisotropy affects how dopants are integrated, how defects propagate, and how surfaces respond to processing. Crystallographic orientation has increasingly been treated as a design parameter in three-dimensional device structures. Selecting an appropriate orientation may reduce defect density or improve dopant incorporation compared with more conventional planes [41,42,43].

Growth parameters play a key role when diamond is deposited on patterned substrates. By carefully choosing process conditions, it is possible to promote lateral overgrowth and tailor the exposed facets to match specific device requirements, for example, by selecting orientations that facilitate dopant incorporation or minimize etch damage [44,45]. When diamond is homoepitaxially grown on (100)-oriented patterned substrates for very low methane concentrations, the growth rate along the [100] direction becomes relatively slow. Lateral facets then expand and can dominate the final morphology [46]. Introducing dopants during growth tends to further reduce the growth rate, which can be exploited to form selectively thin doped layers with high thickness control [47].

There is also growing interest in achieving partial or full planarization during growth. Some degree of planarization may occur naturally, but it can be enhanced by tuning process parameters to favor lateral over vertical growth. This approach is particularly relevant for addressing long-standing issues in diamond device technology, such as etch-induced damage, non-uniform doping profiles, and defect propagation from underlying layers. Consequently, growth conditions and crystal orientation are increasingly considered together with device design when optimizing diamond electronic structures [48].

**Figure 3:**
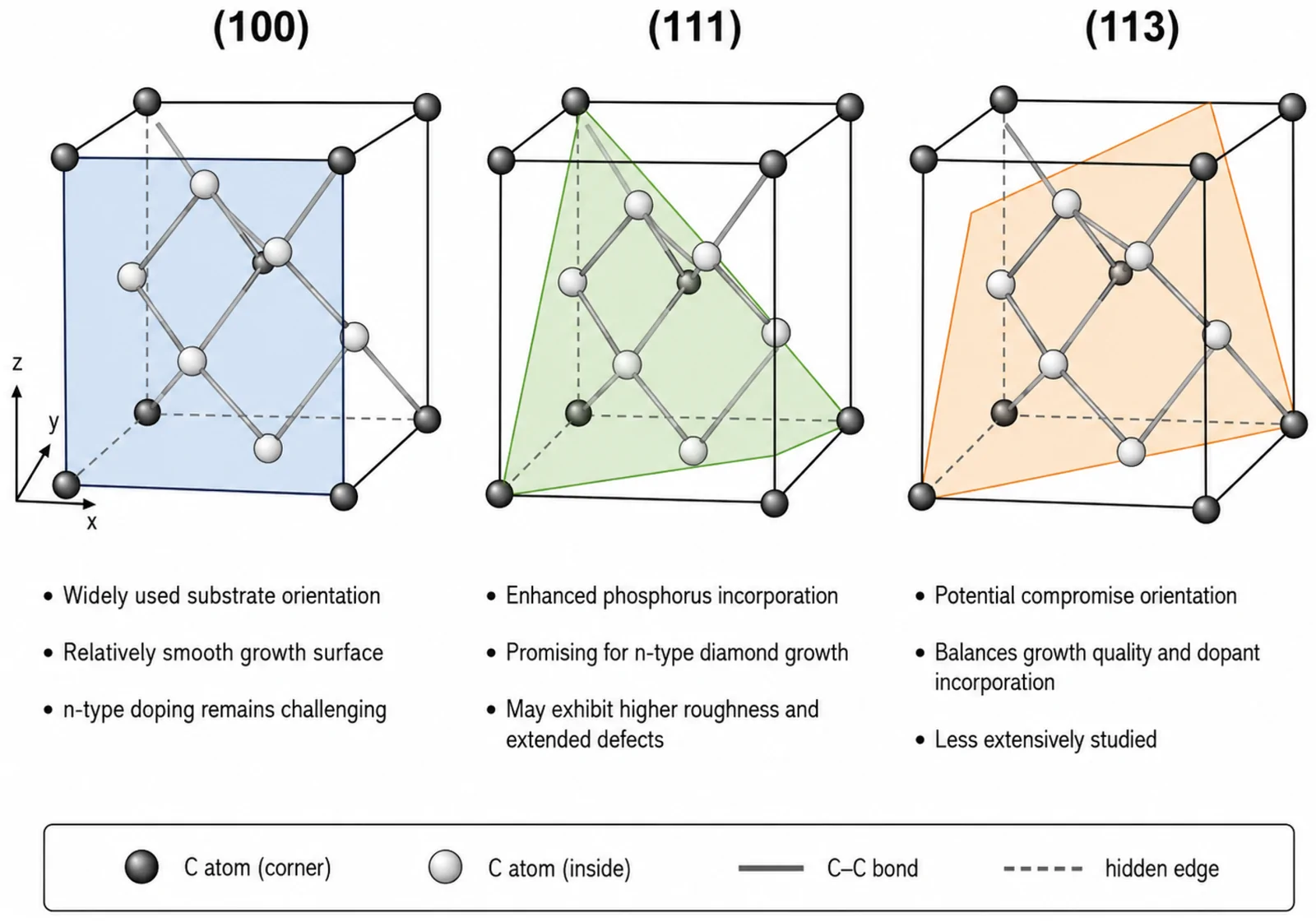
Schematic comparison of the (100), (111), and (113) diamond crystal orientations and their representative growth- and doping-related characteristics.

The role of crystallographic orientation remains an active area of study because it affects growth behavior, dopant incorporation, surface quality, and device performance. The (100) orientation is widely used, but it is not always ideal for doped layers. In particular, high-quality n-type diamond on (100) surfaces is still difficult because donor impurities are hard to incorporate and activate. The (111) orientation can make phosphorus incorporation easier, so it is often used for n-type diamond. At the same time, (111) growth may lead to more extended defects and higher surface roughness [49]. Other orientations, such as (113), have been explored as possible compromises between dopant incorporation, growth control, and surface quality. They may offer better control of doping and growth rate, but they are still less studied and need more systematic evaluation [50]. These orientation-dependent differences also have direct implications for device performance. For example, an oxygen-terminated deep-depletion MOSFET fabricated on (100) diamond exhibited a hole mobility of $1000\pm 200\;{\mathrm{cm}}^{2}\;V^{-1}\;s^{-1}$ and a breakdown voltage of approximately 200V, although its on-state current remained low because of interface-related limitations [51,52]. By comparison, the improved phosphorus incorporation associated with (111) diamond has enabled the formation of n-type body regions for planar p-channel MOSFETs, but the reported devices still showed low drain current and cycling instability [53]. These examples indicate that crystallographic orientation can influence channel transport, junction formation, breakdown behavior, and reliability through its effects on dopant incorporation, surface morphology, and interface quality. Direct comparisons should nevertheless be made cautiously because the reported devices differ in doping concentration, gate stack, geometry, and processing conditions. Figure 3 schematically compares the (100), (111), and (113) diamond orientations and summarizes their main growth, doping, and surface-quality characteristics.

Thus, the same bonding and crystal structure that give diamond its attractive properties also contribute to the difficulty of growth and doping control. Orientation choice can affect defect density, dopant incorporation, surface quality, and device performance. At present, no single orientation gives the best result in every category. A substrate that is good for doping may not be the best for surface smoothness or low defect density. For this reason, substrate orientation has to be chosen based on the device type and target application. In high-voltage and high-power devices, extended defects such as dislocations and stacking faults are especially important because they can increase the local electric field and cause early breakdown.

### 2.3. Doping and Defects

Intrinsic diamond is highly insulating because of its wide bandgap of about 5.47 eV. In a bandgap this large, common dopants have much higher ionization energies than they do in narrow-bandgap semiconductors [54]. Many dopant atoms therefore remain inactive at room temperature. Carrier transport can then be limited by incomplete dopant ionization or carrier freeze-out. For this reason, efficient doping is still one of the main challenges in diamond electronics [49].

For device applications, dopant incorporation alone is insufficient. The material must also provide a stable free-carrier concentration at the operating temperature. This is difficult in diamond because of its strong covalent bonding and high mechanical hardness. Ion implantation can introduce dopants into diamond, but it often produces shallow doped regions, usually only a few tens of nanometers deep. It also creates lattice damage, which must be reduced through high-temperature annealing [50]. Thermal diffusion has been investigated as an alternative route and has enabled the demonstration of diamond p–n diodes, but the technique is not yet established as a robust and reproducible process for complex device structures [55]. In general, introducing dopants while minimizing defect formation requires careful control of growth conditions and dopant concentrations. Techniques such as cathodoluminescence are widely used to analyze the effects of doping and to monitor changes in band structure, including subtle shifts related to the purity and isotopic composition (^12^C vs. ^13^C) of the diamond [56].

For intentional doping, boron is the most commonly used acceptor for p-type diamond, whereas nitrogen and phosphorus are the primary candidates for n-type material [57]. Even so, achieving high free-carrier concentrations at room temperature remains difficult, and this limitation is one of the reasons why diamond devices have not yet reached their theoretical performance potential. A large body of work has examined both heavily boron-doped and phosphorus-doped diamond [58,59,60,61], and experimental models have been developed to estimate impurity content across a wide doping range. Boron has an acceptor activation energy of about 0.37 eV, which is high compared with shallow dopants in Si or GaN but still sufficiently low to be useful as a p-type dopant. Because this activation energy is relatively large, only a fraction of boron dopants are ionized at room temperature, and the hole concentration increases significantly with temperature. Since boron has one fewer valence electron than carbon and a relatively small atomic radius, it can be incorporated into the diamond lattice with comparatively modest lattice distortion, where it acts as an acceptor and generates hole-conducting p-type material [62]. During chemical vapor deposition (CVD) growth, low to moderate boron concentrations can be achieved with reasonable control, but growing thick, highly doped p-type layers without compromising crystalline quality remains challenging [58].

Realizing high-quality n-type diamond is even more problematic. Practical n-type conductivity in diamond is not readily obtained and generally requires synthetic doping approaches [63]. Nitrogen and phosphorus have both been explored as donor impurities, but they introduce relatively deep donor levels. Nitrogen is generally regarded as a deep donor in diamond and therefore acts primarily as a compensating defect rather than a useful donor for practical electron conduction. Phosphorus, by contrast, has a donor activation energy on the order of 0.6 eV, which significantly limits the fraction of ionized donors at room temperature. Under these conditions, the effective electron concentration is strongly reduced. Compensation among donors, acceptors, and unintended impurities therefore becomes a major factor controlling carrier density, mobility, and resistivity [58]. Compensation between donors, acceptors, and residual impurities can drastically reduce the free carrier concentration even when the total dopant concentration is high. Phosphorus incorporation is further hindered by its larger covalent radius, which makes lattice substitution more difficult [61,64]. The strong carbon–carbon bonding and the size mismatch between phosphorus and carbon also limit substitutional incorporation and can introduce lattice distortion or additional defects at higher doping levels. Moreover, the deep donor level keeps a large fraction of incorporated phosphorus electrically inactive at room temperature, so increasing the dopant concentration does not produce a proportional increase in free-electron density. Very heavy doping may reduce resistivity through impurity-band or hopping conduction, but this generally involves a trade-off with crystal quality and carrier mobility. Phosphorus-doped (111)-oriented diamond was first reported in 1997 [65], and comparable doping of (100)-oriented diamond followed in 2005 [66]; however, achieving both high incorporation efficiency and good crystal quality simultaneously is still an open issue. Sulfur has also been investigated as a potential donor species. However, its behavior remains controversial because n-type characteristics have been observed only under certain conditions, and the underlying mechanism is not yet fully understood [67].

Defects in diamond layers, both macroscopic and microscopic, can strongly affect device performance. Non-epitaxial crystallites that can appear in nominally homoepitaxial films may degrade the operation of power and RF devices by locally distorting electric fields or acting as leakage paths [31]. Macroscopic defects visible under optical inspection can lead to premature breakdown or excessive leakage in Schottky diodes, MESFETs, MOSFETs, and related structures [32,33,34]. At the microscopic level, point defects, dislocations, and impurity complexes introduce states within the bandgap that influence carrier concentration, scattering, recombination, and thereby affect conductivity and mobility [68]. In high-voltage devices, these defects can also act as localized field-enhancement sites that trigger premature breakdown or increase leakage current. For high-performance diamond electronics, it is therefore not sufficient to optimize nominal dopant levels alone. Controlling the formation, distribution, and evolution of defects is equally important for achieving reliable, reproducible device characteristics. Ongoing research therefore focuses on improving dopant activation, reducing compensation, and exploring alternative doping strategies such as delta-doping and high-concentration epitaxial doping to enhance carrier densities in diamond.

## 3. Physical Properties of Diamond

### 3.1. Temperature-Dependent Electrical Characteristics

In silicon power devices, dopants are usually nearly fully ionized at room temperature. At higher temperature, their performance can degrade because leakage current increases and the blocking capability becomes harder to maintain. Boron-doped diamond can show a different temperature response. In p-type diamond, the normalized specific resistance decreases strongly as temperature increases, unlike the more familiar trend observed in many conventional semiconductors [69].

This response is mainly linked to boron ionization in diamond. Boron acceptors have a relatively large activation energy, about 0.37 eV. At room temperature, only part of the boron is ionized, so the free-hole concentration is limited. This can increase the resistance of boron-doped regions and may limit on-state performance in some diamond device structures. When temperature increases, more boron atoms become ionized. More holes are then available for conduction, and the resistivity decreases. This decrease is useful, but it does not continue without limit. At higher temperatures, mobility loss and parasitic effects may reduce part of the benefit.

This temperature dependence should not be generalized to all diamond FETs. In p-channel devices based on a two-dimensional hole gas at the hydrogen-terminated diamond surface, the channel is formed by surface transfer doping rather than by bulk dopant ionization. If the surface is well passivated, for example, with an ALD-grown dielectric, the sheet hole density can remain nearly constant with temperature [70]. Because of this, the channel conductivity may show only weak temperature dependence when the surface charge is stabilized. Long-term stability of the surface termination and dielectric interface is still a concern.

Diamond devices can therefore show different temperature responses depending on how the channel is formed. In boron-doped material, conduction may improve with temperature because more acceptors become ionized. In hydrogen-terminated surface-channel devices, the behavior is controlled more by the stability of the surface charge and dielectric interface. This distinction is important for designing diamond power and RF devices [69].

### 3.2. Negative Electron Affinity (NEA) Characteristics

Electron affinity is a key surface property of diamond because it controls electron emission and strongly depends on surface termination. Electron affinity is the energy difference between the conduction-band minimum (CBM) and the vacuum level. In most semiconductors, this value is positive, so an electron at the CBM still needs extra energy to escape into vacuum. Hydrogen-terminated diamond can exhibit negative electron affinity (NEA). In this case, the vacuum level lies below the CBM, and electrons near the conduction band edge can be emitted into vacuum with little or no surface barrier. This effect was first reported by Cui et al. in 1998 [71].

NEA in hydrogen-terminated diamond is commonly attributed to C–H surface dipoles that lower the vacuum level relative to the conduction-band minimum. Oxidation can reverse or modify the surface dipole, converting the surface from negative to positive electron affinity. For this reason, NEA depends strongly on surface termination. This is important for devices, since the surface chemistry must remain stable during fabrication and operation.

Hydrogen termination is also central to the formation of a high-density hole channel near the diamond surface. Under ambient conditions, or when suitable surface acceptors are present, hydrogen-terminated diamond can support sheet hole concentrations on the order of 10^13^ cm^−2^ [72,73,74,75]. These holes do not come from ionized dopants in the diamond bulk. They form through surface transfer doping, where electrons move from the diamond valence band into acceptor states near the surface and leave mobile holes behind.

This mechanism allows p-channel MOSFETs to be formed without boron doping in the channel. Compared with conventional doped-channel devices, these two-dimensional hole gas (2DHG)-based MOSFETs may offer advantages for high-frequency and some high-temperature applications. Their performance still depends strongly on the surface interface and on the quality of the gate dielectric on hydrogen-terminated diamond [76,77,78,79]. In practice, threshold-voltage stability, adsorbate control, and dielectric compatibility remain the main issues.

Negative electron affinity also makes hydrogen-terminated diamond useful for cold-cathode emission. Because electrons near the conduction-band minimum face little or no emission barrier, the surface can show efficient electron emission with relatively modest electric fields. This behavior is unusual among established semiconductors and is still studied for vacuum microelectronics, electron sources, and related applications [80]. Even so, emission uniformity and long-term surface stability must be controlled for reliable device operation.

### 3.3. Deep Depletion Characteristics

In metal–oxide–semiconductor (MOS) structures, the surface condition changes with the applied gate bias. Depending on the bias, the semiconductor surface may enter accumulation, depletion, or inversion. When the gate voltage is swept slowly, minority carriers usually have enough time to be generated. Once the surface potential reaches the required level, these carriers can form an inversion layer. The situation is different with a fast voltage sweep, such as a pulsed gate bias. In diamond, the intrinsic carrier concentration is extremely low, so minority-carrier generation is very slow. The electric field can change faster than the carriers can respond. Instead of forming an inversion layer immediately, the depletion region continues to expand. This non-equilibrium condition is known as deep depletion [81].

In this regime, charge neutrality is temporarily supported mainly by ionized dopants rather than by thermally generated minority carriers. As a result, the space-charge region extends farther into the substrate. Under deep-depletion conditions, the depletion width increases with gate voltage until it approaches a maximum value set by the doping concentration, temperature, and material parameters [82]. If the voltage sweep is slowed, or if the device is allowed enough time to relax, the system can return toward equilibrium. An inversion layer may then form, and the depletion width reduces back toward its steady-state value.

For the same temperature and doping level, wider-bandgap materials generally allow a larger maximum depletion width. This occurs because lower intrinsic carrier concentration delays inversion-layer formation. Diamond, with a bandgap of about 5.47 eV, can therefore sustain unusually wide depletion regions before inversion develops. In practical diamond MOS devices, inversion may be delayed or strongly suppressed because minority carriers are generated so slowly. This behavior may also contribute to the relatively high threshold voltages reported in some diamond FETs, although interface charge, surface chemistry, traps, and gate-stack quality can also play major roles. For this reason, design assumptions taken directly from silicon MOS technology do not always transfer cleanly to diamond.

Related wide-depletion behavior is also important in other device classes where depletion width controls the device response, including Schottky diodes, heterojunction bipolar transistors (HBTs), varactors, and avalanche photodiodes. In these devices, a wide depletion region can be useful, especially when combined with diamond’s high breakdown field. It supports high-voltage and high-power operation. The same feature can also make the device harder to control. Threshold voltage may shift, and the transient response can become more complicated. For this reason, transient modeling and interface characterization are needed when these devices are designed for circuits.

In diamond devices, deep depletion should be treated as both a design opportunity and a device-control challenge. It can help support high electric fields across a wide depletion region. The challenge is to design around its side effects, including delayed inversion, relatively high threshold voltage, and non-equilibrium charge behavior in power and RF devices.

### 3.4. Breakdown Field and Voltage

Breakdown voltage is a key parameter for power semiconductors because it is closely related to the critical electric field. In diamond, this is one of the main material benefits. Given that the bandgap in diamond is about 5.47 eV, the theoretical critical electric field of diamond is often estimated to exceed 10 MV cm^−1^. For comparison, typical critical fields are approximately ∼3 MV cm^−1^ for SiC and ∼3.5 MV cm^−1^ for GaN [83,84]. High electric fields in diamond are explained by the presence of the large bandgap, which implies a greater energy required for impact ionization to become significant and cause avalanche breakdown. In practical devices, however, the measured breakdown field is often much lower because of materials and processing limitations.

Processed diamond devices often show breakdown fields of only a few MV cm^−1^. This is primarily because of defects in the crystals, field enhancement at contact regions, and edge termination effects. These limitations do not necessarily reflect the fundamental properties of diamond. Rather, they indicate that present device performance is still strongly limited by material quality and processing control.

**Figure 4:**
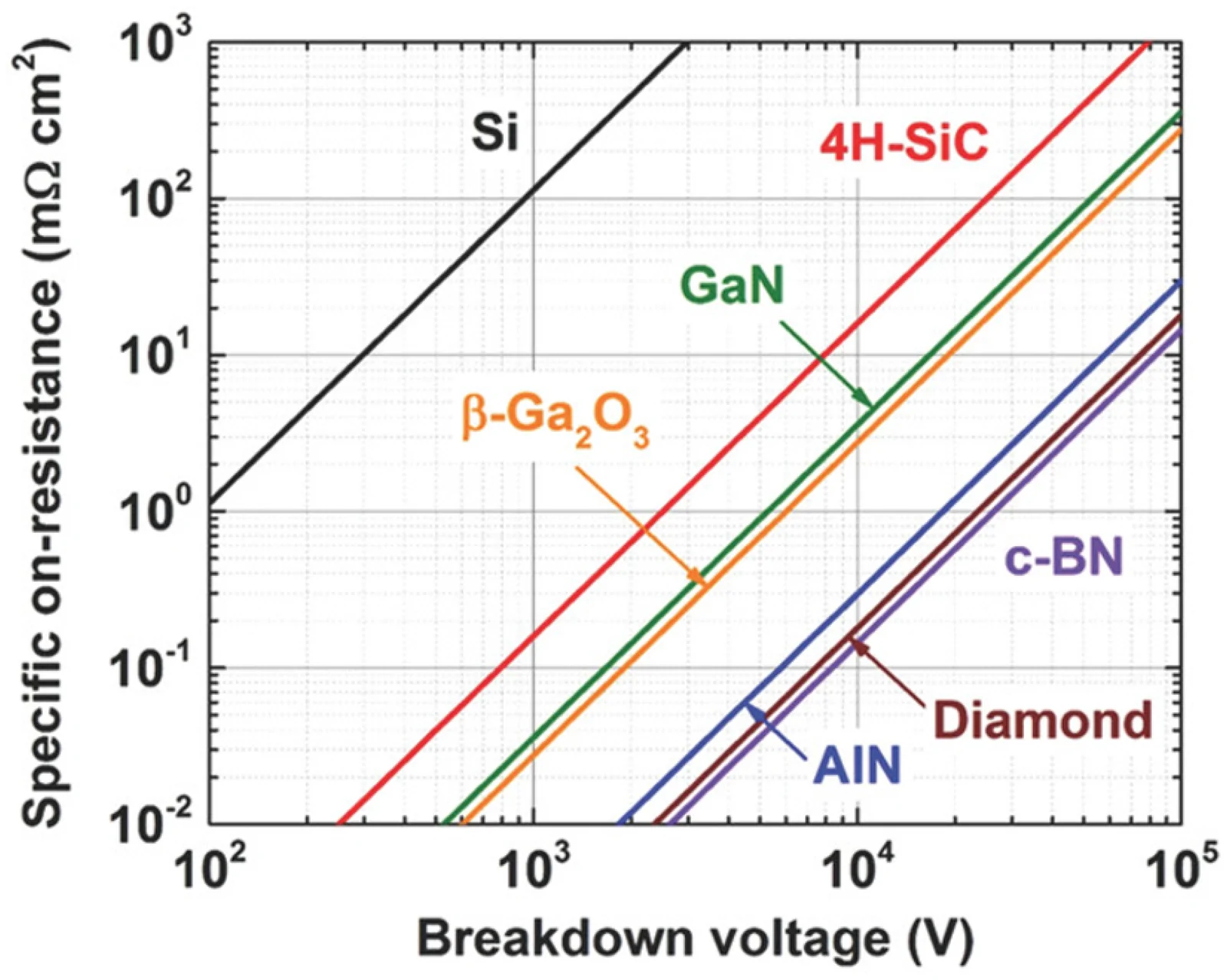
Specific on-resistance versus breakdown voltage for selected semiconductors at room temperature. Adapted from [12].

The critical field is relevant for unipolar devices, influencing their drift layer thickness, doping concentration, and blocking voltage. An increase in the breakdown field means that the drift layer thickness can be reduced for a certain level of voltage, or the blocking voltage can be increased for a constant drift layer thickness. Under the assumption of ideal operation of a unipolar device, the specific on-resistance, $R_{\mathrm{on},\mathrm{sp}}$, scales approximately with $1/E_{c}^{3}$. This trend explains why diamond is expected to offer a much lower theoretical on-resistance at high breakdown voltages than conventional WBG semiconductors, as illustrated in Figure 4.

Analogous problems arise in diodes, FETs, and other junction structures, although the constraining factors do not have to be exactly the same in all situations. The breakdown voltage is governed by the critical electric field, but it is also affected by the substrate material quality, dopant profile, contacts, and electrode configuration. Termination is particularly crucial, since the field plates, guard rings, and junction termination all need to distribute the electric field without causing additional high-field locations. In many cases, field management is as important as improving the diamond bulk quality.

Although diamond has an exceptionally high intrinsic breakdown field, practical devices remain limited by defects, edge termination, and electric-field crowding. To exploit this intrinsic advantage more effectively, better control of device structure and processing is required.

## 4. Optoelectronic Properties and UV Photodetection

Diamond’s indirect ultrawide bandgap of about 5.47 eV gives it an absorption edge near 225 nm, making it naturally suited for deep-ultraviolet detection. Photons with longer wavelengths have energies below this bandgap, so intrinsic diamond does not absorb them strongly. The cutoff is in the deep-UV/solar-blind range, although the intrinsic diamond edge is closer to 225 nm than to 280 nm. In practical UV detectors, this helps reduce visible and infrared response without using external optical filters [81].

Diamond also has strong radiation hardness and good thermal stability. These properties make it useful in harsh environments, including ionizing-radiation fields, high-temperature systems, and vacuum-based devices. For this reason, diamond has been studied for UV photodetection along with other wide-bandgap materials, such as cubic BN [82], GaN [85,86,87], Al_*x*_Ga_1−*x*_N [88,89,90], ZnS [91,92], ZnO [93,94], Zn_*x*_Mg_1−*x*_O [95,96], SiC [97,98], and more recently, β-Ga_2_O_3_ [99,100,101,102,103].

Several diamond UV detector structures have been reported. These include photoconductors, Schottky barrier photodiodes, metal–semiconductor–metal (MSM) detectors, metal–insulator–semiconductor (MIS) devices, p–n and π–n photodiodes, and field-effect or bipolar phototransistors [104]. Their responsivity, dark current, and response speed can vary widely. These differences usually depend on surface termination, defect density, electrode layout, and dielectric integration.

The operating mechanism depends on whether the device is mainly controlled by bulk photocarrier transport or by surface transfer doping at a hydrogen-terminated surface. In these devices, the hydrogen-terminated surface, which is associated with NEA behavior, can also support a two-dimensional hole gas. Under UV illumination, this surface channel can contribute to photoconductive gain. The gain is usually linked to photogenerated carriers changing the surface charge balance, although the same process may also affect response speed and long-term stability.

Device performance is frequently limited by contact resistance, defect-related trap states, and interface quality rather than by diamond’s intrinsic absorption edge alone. Work continues on improving responsivity and reducing dark current through refinement of surface preparation, passivation, and heterostructure design. Although UV photodetectors are not the central focus of this review, they illustrate how the same surface, defect, and contact issues that limit diamond power devices also affect other diamond electronic platforms.

## 5. Figures of Merit

Power semiconductor devices are influenced by several types of losses: conduction losses in the ON-state, switching losses during transitions, and leakage losses in the OFF-state. In principle, a single figure of merit (FOM) that fully characterizes power-device performance would need to incorporate all of these while also reflecting system-level constraints such as thermal management, gate driving, electromagnetic compatibility (EMC), reliability, and cost. In practice, this is not feasible. Switching losses, for example, depend not only on the device itself but also on the driver circuit, the converter topology, for example, hard or soft switching, and parasitic inductances and capacitances introduced by packaging and layout.

A widely used FOM for unipolar power devices is Baliga’s figure of merit (BFOM), derived from the specific on-state resistance $R_{\mathrm{on},\mathrm{sp}}$ as defined in [105] and Equation (1). This metric is derived under the assumption of bulk conduction through a uniformly doped drift region with fully ionized dopants. Equation (2) expresses $R_{\mathrm{on},\mathrm{sp}}$ in terms of BFOM and breakdown voltage (BV) and captures the usual trade-off between low conduction loss and high blocking capability. The derivation assumes complete ionization of dopants and bulk conduction in the drift region. These assumptions are often not valid for diamond power devices. In bulk diamond, incomplete ionization of dopants substantially modifies the relation between doping, carrier density, and $R_{\mathrm{on},\mathrm{sp}}$, while in 2DHG-based devices the relevant quantities are sheet carrier density and two-dimensional (2D) mobility rather than bulk doping. In particular, the high activation energy of common dopants such as boron and phosphorus results in partial ionization at room temperature, which increases the effective resistivity of the drift region. Conventional bulk power-device scaling rules do not transfer directly to surface-channel diamond devices. In this case, Equation (2) has to be used with care, since the specific ON-resistance, $R_{\mathrm{on},\mathrm{sp}}$, cannot always be estimated from BFOM alone. If conventional unipolar FOMs are applied without considering diamond-specific material and device limits, they may make diamond devices appear more favorable than they are in practice.

For this reason, $R_{\mathrm{on},\mathrm{sp}}$ is often used as a more direct figure of merit when devices are compared within a similar breakdown-voltage range. In experiments, the ON-state resistance is usually extracted from pulsed I–V measurements or estimated using analytical and numerical models. The area used for normalization may refer only to the active region, or it may also include the termination region, depending on the convention used. Likewise, the breakdown voltage, BV, may be measured directly or calculated under stated assumptions.

Direct comparison among reported diamond and other WBG and UWBG devices remains difficult because $R_{\mathrm{on},\mathrm{sp}}$, BV, active area, termination area, measurement temperature, and breakdown criteria are not reported using consistent conventions. Switching losses are often excluded, active-area definitions and breakdown criteria vary, and basic $R_{\mathrm{on},\mathrm{sp}}$ values do not capture thermal effects. The temperatures at which $R_{\mathrm{on}}$ and BV are measured should therefore be reported because both parameters are temperature-dependent [20].

(1) $$\mathrm{BFOM}=\varepsilon \mu E_{c}^{3}$$

(2) $$R_{\mathrm{on},\mathrm{sp}}=\frac{4BV^{2}}{\varepsilon \mu E_{c}^{3}}=\frac{4BV^{2}}{\mathrm{BFOM}}$$

Here, ε denotes the semiconductor permittivity, μ is the carrier mobility, $E_{c}$ is the critical electric field, BV is the breakdown voltage, and $R_{\mathrm{on},\mathrm{sp}}$ is the specific on-resistance.

Switching-related FOMs, such as $R_{\mathrm{on}}\cdot Q_{g}$, $R_{\mathrm{on}}\cdot Q_{\mathrm{gd}}$, and $R_{\mathrm{on}}\cdot Q_{\mathrm{oss}}$, related to the switching characteristics, are also employed to evaluate the performance of devices [106,107,108]. In these expressions, $Q_{g}$ is the total gate charge, $Q_{\mathrm{gd}}$ is the gate–drain charge, $Q_{\mathrm{oss}}$ is the output charge associated with the output capacitance, and $Q_{\mathrm{gs}}$ is the gate–source charge. These FOMs combine ON resistance with capacitive charging contributions and are suitable for the evaluation of fast-switching unipolar devices. However, their use in the case of diamond devices is more difficult due to the limited size of the active region and difficulties in capacitive characterization of the devices. Such FOMs are most applicable to unipolar devices, where minority-carrier storage is negligible. For diamond MOSFETs, $R_{\mathrm{on}}Q_{g}$ and $R_{\mathrm{on}}Q_{\mathrm{gd}}$ describe the trade-off between conduction resistance and gate-charge-related switching performance, whereas $R_{\mathrm{on}}Q_{\mathrm{oss}}$ reflects the influence of output charge [106,107,108]. These FOMs are most relevant to unipolar, gate-controlled devices. By contrast, bipolar diamond p–i–n diodes should also be evaluated using reverse-recovery time and stored charge, which are not captured by gate-charge-based FOMs [109].

For diamond power devices, optimization should not depend on one conventional FOM. A more useful assessment needs to consider conduction loss and switching behavior together, while also accounting for heat dissipation, breakdown design, and the actual device geometry. Reducing $R_{\mathrm{on},\mathrm{sp}}$ alone is insufficient. Total gate charge $Q_{g}$, gate–drain charge $Q_{\mathrm{gd}}$, and output charge $Q_{\mathrm{oss}}$ should also be characterized and minimized where possible. The Miller ratio between $Q_{\mathrm{gd}}$ and $Q_{\mathrm{gs}}$ should likewise be controlled. Other relevant quantities include robustness to high dV/dt and dI/dt, maximum turn-on and turn-off speeds, and gate leakage over the intended operating temperature range [20]. These parameters together provide a more complete basis for assessing and comparing diamond-based power devices. Future benchmarking efforts would benefit from standardized reporting practices so that results from different diamond device architectures can be compared more consistently. Although diamond has exceptionally high bulk thermal conductivity, practical heat removal is also governed by the complete device and package thermal path. Thermal-boundary resistance at the diamond/dielectric, metal-contact, die-attach, and substrate interfaces can limit heat spreading, particularly in small-area high-current devices. Packaging materials must also withstand the intended temperature, electric field, and thermal-cycling conditions. Consequently, junction-temperature measurements and package-level thermal resistance are needed in addition to bulk material properties when evaluating diamond power devices.

## 6. Diamond Electronic Devices

### 6.1. Microfabrication Techniques of Diamond Devices

Diamond’s extreme mechanical hardness, chemical stability, wide bandgap, and high thermal conductivity make it attractive for electronic and microelectromechanical applications, but these same properties also complicate device processing. Device fabrication, however, is constrained by substrate size, defect density, and the difficulty of patterning and thinning single-crystalline diamond (SCD). SCD devices are generally produced by homoepitaxial or heteroepitaxial growth on pre-existing diamond substrates, which remain limited in both area and cost [49]. In addition, the absence of well-established wet etching processes and the chemical inertness of diamond make conventional semiconductor processing techniques difficult to apply.

Fabrication typically involves two stages: (1) forming a thin diamond device layer or membrane and (2) processing that layer into a functional electronic structure. Several micromachining approaches have been developed for this purpose, including thinning by dry etching, angle etching, ion-implantation liftoff, and focused ion beam (FIB) milling [110,111].

Thin-down processing typically begins with bulk diamond plates that are tens of micrometers thick. The material is thinned by dry etching, commonly using capacitively coupled reactive ion etching (RIE), down to a few hundred nanometers [112]. Oxygen-based plasma chemistries are most commonly used because oxygen reacts with carbon to form volatile CO and CO_2_ species. While straightforward, this process is limited by starting wafer size and by variations in the initial thickness, which can carry through to the final device layer unless monitored and controlled [113]. Surface roughening and plasma-induced damage may also require additional post-processing.

Angle etching follows a different process. The diamond is first patterned with an etch mask and etched vertically. After the target etch depth is reached, the sample is tilted so that the ion beam strikes the diamond surface at an oblique angle. This angled exposure forms an undercut and can be used to release suspended diamond structures [114,115]. The main advantage of this process is that microstructures can be fabricated free-standing without any preliminary substrate thinning. However, the method also limits design flexibility. Once the angle of etching and lateral dimension are chosen, the thickness of the resulting membrane is constrained by the tilt angle.

Ion-implantation liftoff follows a different principle. First, ion irradiation is used to create a buried damaged layer inside the diamond [116,117,118]. Subsequent annealing converts the damaged region into a graphitized layer, which can then be selectively removed. This process releases a diamond membrane. The method provides some advantages in comparison with direct thinning approaches in terms of obtaining the membrane with a homogeneous thickness; however, precise control of the implantation is required.

Focused ion beam milling is another method of fabricating thin-film diamond structures. Using ions such as Ga^+^, FIB enables direct patterning of membranes and microstructures with sub-micron resolution [119,120]. Its advantage lies in local structuring, small structures, and nonplanar shapes because no masks are needed. On the other hand, ion irradiation can introduce surface damage and implanted Ga-related defects. Residual Ga^+^ incorporation and other defects associated with ion implantation will persist without further annealing or cleaning treatments. This method is also rather time-consuming, which makes it less suitable for large-scale production. Some researchers have attempted to overcome these limitations by combining FIB with electron beam etching [121,122,123,124].

Together, these techniques offer different approaches to fabricating thin or suspended diamond structures. No single approach simultaneously provides high throughput, low damage, high dimensional control, and large-area scalability. The trade-off between uniformity, minimization of damage, precision, and machining speed must still be considered. The trade-off takes on particular significance when fabricating larger-area devices that operate at high voltages because it is important that the active diamond film layer is not damaged during machining and that the machining process can be repeated reliably. At present, fabrication maturity remains one of the main barriers separating diamond devices from more established WBG technologies. These considerations show that diamond’s theoretical material advantages cannot be evaluated independently of device architecture, fabrication quality, and measurement conditions. The following section therefore examines how these material-level opportunities and limitations are reflected in reported diamond electronic devices.

### 6.2. Diamond Diodes

Diamond power diodes have been reported in both unipolar and bipolar forms. Diamond diodes show strong progress in blocking voltage and current density, but reproducibility, n-type doping, contacts, and edge termination still limit practical use. The main examples are Schottky-type diodes, p–n diodes, and p–i–n diodes. This section reviews the main diode structures reported so far and compares their representative performance. Table 3 summarizes representative results reported for diamond diodes, highlighting key performance metrics such as breakdown voltage, current density, and specific on-resistance. Although these results demonstrate substantial progress, the values summarized in Table 3 should not be interpreted as a direct ranking of device architectures. Breakdown voltage, specific on-resistance, and current density depend strongly on drift-layer design, active-area definition, edge termination, material quality, temperature, and measurement criteria. In addition, many studies report the best-performing small-area device without wafer-level statistics or long-term reliability data. The variation among reported results therefore reflects differences in device geometry, fabrication, and measurement conditions, while reproducibility remains an important challenge.

#### 6.2.1. Schottky Diodes and Metal/Diamond Contacts

Several types of diamond-based Schottky diodes have been demonstrated, including conventional Schottky barrier diodes (SBDs), junction barrier Schottky diodes (JBSDs), metal–intrinsic–p (M–i–P) diodes, and Schottky p–n diodes (SPNDs), which combine characteristics of both SBDs and p–n diodes [140]. SPNDs offer a lower on-voltage than conventional p–n diodes and a lower specific on-resistance and improved reverse-blocking capability compared with standard SBDs. These devices combine the fast switching characteristics of Schottky diodes with the improved reverse blocking capability of p–n junctions. They have been reported to sustain forward current densities up to 10^4^ A/cm^2^ at 7 V with a low differential on-resistance of ∼10^−5^ Ω·cm^2^ over a wide temperature range from 295 K to 600 K [141]. These results highlight the potential of hybrid diode concepts to balance conduction and blocking performance.

M–i–P diodes, on the other hand, have been extensively studied for their ability to achieve high breakdown voltages with relatively thin drift regions, benefiting from the large critical electric field of diamond [126,142]. Zhao et al. fabricated a vertical M–i–P diamond SBD with different drift-layer thicknesses by growing undoped single-crystal diamond on HPHT boron-doped diamond substrates and depositing Mo/Ni/Au as the Schottky electrode on the drift layer with Ti/Au as the back ohmic contact. The maximum forward current density reached 7570 A/cm^2^ at −10 V, while the minimum reverse current density was as low as 100 pA/cm^2^ at 10 V. The rectification ratio and breakdown electric field reached ∼10^12^ and 4.2 MV/cm, respectively [126].

Hall measurements have confirmed the successful incorporation of n-type dopants in diamond [132]. This achievement enabled the fabrication of Schottky PiN diodes with promising electrical characteristics. These devices exhibit forward current densities exceeding 300 A/cm^2^ at only 4 V of forward bias and breakdown voltages above 500 V, despite having a relatively thin 3.5 μm drift layer. They also show a low turn-on voltage, high forward current density, and an ideality factor close to unity, with the blocking voltage scaling with the thickness of the intrinsic layer [131]. Furthermore, Takeuchi et al. demonstrated a 10 kV vacuum switch with a power-transmission efficiency of 73% using a diamond PiN diode [143], highlighting the potential of diamond junction devices for high-power switching applications [131]. Even so, reproducible n-type doping remains a major bottleneck for broader deployment of bipolar diamond diodes.

The high-power capability of diamond devices was initially demonstrated using SBDs with boron-doped p-type layers. Diamond SBDs exhibit impressive characteristics, including breakdown fields exceeding 7 MV/cm even without edge termination, low leakage current, short turn-off transients, and small reverse-recovery charge, even at elevated temperatures. Breakdown voltages approaching the multikilovolt range with relatively low on-resistance and stable high-temperature operation have been reported [144,145,146,147,148]. Among these results, Volpe et al. reported diamond Schottky diodes with breakdown voltages up to 10 kV, while two-dimensional modeling indicated an electric field of approximately 7.7MV/cm at the center of the device [145]. Such high-voltage SBDs commonly employ p-type diamond layers as drift and contact regions because their doping concentration, 10^15^–10^22^ cm^−3^, can be controlled reliably [22,149], while hole mobility is significantly higher than electron mobility in n-type layers [150].

The quality of the metal/diamond interface is crucial for achieving high-performance SBDs. The Schottky barrier height depends strongly on both the metal work function and the surface termination of the diamond. Surface termination, as in other wide-bandgap semiconductors, strongly influences the electrical characteristics of the diamond surface. Oxygen termination is widely used to form stable Schottky contacts with high Schottky barrier heights (SBHs). Typical oxygen-termination methods include acid mixture treatments, oxygen plasma, and ultraviolet treatment [151,152,153,154,155]. Although many metals have been investigated as Schottky contacts [140,156,157,158,159], high-melting-point metals such as Mo, Pt, Ru, and Zr have yielded some of the strongest reported high-voltage SBD performance [160,161,162]. Depending on the metal species and surface treatment, the SBH can be tuned between ∼1.2 and 3.4 eV [163]. Such large barrier heights are advantageous for achieving low reverse leakage currents in high-voltage devices. In contrast, n-type diamond layers remain highly resistive at room temperature because of the large donor activation energies, 0.57 eV for P and 1.7 eV for N. In addition, strong Fermi-level pinning leads to large, metal-independent SBH values, 4.3–4.5 eV [164,165,166,167]. This combination continues to hinder the realization of low-resistance n-type Schottky contacts.

More recently, interfacial and edge-termination engineering has become a central lever to approach diamond’s theoretical breakdown field. Vertical SBDs incorporating high-*k* HfO_2_ field plates have demonstrated a substantial improvement in breakdown performance. Increasing the field-plate thickness from 200 to 400 nm raised the breakdown voltage from ≈280 V to ≈314 V and the corresponding breakdown field from ∼1.8 to ∼3.0 MV/cm, with minimal penalty in on-state loss [168]. Design studies using nitrogen-doped field-plate and junction-termination-extension (JTE) concepts likewise predict that appropriately shaped field plates can significantly relax the trade-off between specific on-resistance and blocking voltage in vertical SBDs [169]. These results emphasize that termination design is often as critical as the bulk material itself.

Beyond planar layouts, fluorine-terminated (FT) vertical SBDs have been reported, achieving a turn-on voltage of 1.6 V, a specific on-resistance of 50.2 mΩ·cm^2^, a breakdown voltage of 117 V, and a breakdown field of 3.3 MV/cm for an FT length of 20 μm. The FT termination reduced the turn-on voltage and increased the breakdown field compared with otherwise identical SBDs without FT, illustrating how surface chemistry and lateral device geometry can be co-optimized for high-performance power devices [129].

Pseudo-vertical SBDs further improve the trade-off between conduction and blocking by optimizing drift-layer thickness and cathode geometry. Using an empirical mobility model calibrated to experimental data, one such device achieved a specific on-resistance of 6.8 mΩ·cm^2^, a breakdown voltage of 1190 V, and a figure of merit of 210 MW/cm^2^, while maintaining acceptable forward-voltage drop at current densities around 100 A/cm^2^ [170]. These results confirm that even in relatively compact structures, diamond SBDs can reach Baliga figures of merit comparable to or exceeding those of SiC and GaN devices. However, large-scale manufacturability of these optimized structures has yet to be demonstrated.

In terms of ohmic contacts, titanium (Ti) remains the most widely used material for both p-type and n-type diamond. A typical process consists of Ti deposition followed by annealing in N_2_ or Ar. During annealing, Ti reacts with the diamond surface, for example, through carbide formation, which lowers the contact resistance. For example, a p-type diamond (100)/Ti contact exhibited a specific contact resistance as low as 2.8 × 10^−7^ Ω·cm^2^ after annealing for 60 min at 420 °C in Ar [171].

Vertical-type SBDs using these contact and termination schemes have shown impressive performance, with breakdown voltages in the 1.8–3.7 kV range [147,156,172,173]. Devices with an electrode area of 16 mm^2^ can handle forward currents up to 10 A, and their breakdown field has been measured at ∼7.7 MV/cm [169,172]. The reverse leakage current is generally attributed to thermionic field emission with barrier lowering [173,174]. Abrupt increases in leakage current and reduced breakdown fields are often associated with crystallographic defects originating from the substrate, CVD growth, or device processing [175,176]. A representative vertical Pt-based Schottky diode employed CVD diamond layers on an HPHT diamond substrate with a Ti/Pt/Au ohmic contact [176].

At the same time, lateral architectures have progressed rapidly. Diamond p-type lateral SBDs with Al_2_O_3_ field plates now exhibit rectification ratios >10^7^, peak current densities of 5.39 mA/mm, and a record breakdown voltage of 4.6 kV at a leakage level of only 0.01 mA/mm, demonstrating that field-plate engineering can push lateral devices into the multikilovolt regime [174]. On the vertical side, recent work on SBDs with anodic self-aligned mesa termination has achieved breakdown voltages exceeding 450 V together with specific on-resistance of a few mΩ·cm^2^ and on/off ratios around 10^9^, confirming experimentally that mesa-type terminations can effectively suppress edge field crowding in compact vertical devices [175]. Overall, Schottky-based diamond diodes have shown some of the most mature and competitive results in diamond power electronics, although continued progress in n-type doping, substrate quality, contact resistance, defect control, edge termination, and scalable fabrication remains necessary.

#### 6.2.2. Diamond PN and PiN Junction Devices

Semiconductor p–n and p–i–n (PiN) diodes are fundamental two-terminal structures used for high-power rectification and also form the building blocks of three-terminal devices. In diamond, they have been explored for both power conversion and radiation-energy harvesting. Shimaoka et al. [177] demonstrated a diamond p–n junction betavoltaic cell with high semiconductor conversion efficiency, illustrating the potential of diamond junctions for radiation-driven energy conversion. This result shows that diamond p–n junctions are relevant not only for rectification but also for radiation-tolerant energy-conversion devices.

Dynamic switching behavior has also been investigated in diamond PiN diodes. Traoré et al. [109] evaluated the reverse-recovery behavior of diamond PiN diodes used as free-wheeling elements. The devices commutated from conduction to blocking at ≈850 A/cm^2^ with a reverse-recovery time of ∼150 ns. These results indicate that diamond PiN structures can combine high blocking capability with relatively fast recovery in hard-switched converters. These advantages come at the cost of higher forward on-voltage, set by the 5.47 eV bandgap, and increased stored charge in the drift region. The high forward voltage is primarily a consequence of the large bandgap and the relatively high activation energy of dopants. Therefore, PiN diodes are most attractive in ultra-high-voltage stages where silicon devices are limited by impractically thick drift layers.

Progress in junction formation and doping has also been substantial. Koizumi et al. realized high-quality diamond p–n junctions in 2001 and subsequently developed optimized diamond p–n and PiN UV LEDs [134,178]. Boron and phosphorus remain the standard acceptor and donor species for p-type and n-type layers, respectively. Despite their relatively high activation energies, recent work has demonstrated that extremely high doping levels can be achieved for both p-type and n-type diamond while maintaining good crystal quality. Regarding reverse-blocking properties, a PiN diode with a breakdown voltage of 11.5 kV and rectification ratio of 10^7^ has been reported [133]. This device employed MPCVD growth of undoped and p-doped diamond layers on a p-type diamond substrate, with electrodes on both surfaces; the drift-layer thickness was ∼70 μm and the estimated breakdown field was 1.9 MV cm^−1^ [179]. The introduction of a mesa structure in similar PiN diodes significantly reduced reverse leakage, and both breakdown voltage and breakdown field were found to increase with drift-layer thickness in good agreement with analytical predictions [180]. The highest experimentally reported breakdown field in diamond PiN diodes is on the order of a few MV/cm for a PiN diode with a 2 μm drift layer, and further optimization of terminations, device geometry, and crystal quality may help move measured breakdown fields closer to the theoretical limit of diamond. At present, however, measured values remain well below the intrinsic material limit [133,179].

Diamond p–i–n diodes enabled by highly phosphorus-doped epitaxial layers have also been demonstrated with a breakdown field exceeding 1.25MV/cm [132]. Schottky p–i–n (PiND) structures extend this concept. Dutta et al. reported diamond-based Schottky p–i–n diodes with forward current density >300 A/cm^2^ at 4 V and blocking voltage >500 V, along with low turn-on voltage, an ideality factor near unity, and clear scaling of blocking voltage with intrinsic-layer thickness [131]. These devices highlight that, despite the challenges of deep donors, careful epitaxial growth and contact engineering can yield promising bipolar performance in the sub-kV regime.

Beyond simple planar layouts, more advanced junction-engineering strategies have emerged to exploit diamond’s large breakdown field. Trenched p–n diamond junction diodes have been designed and optimized to improve the trade-off between on-resistance and breakdown voltage [135]. Compared with a beveled reference structure, the trenched p–n design enhances conductance modulation and increases forward current by ∼170%. Simulations indicate that higher n-layer doping reduces series resistance, but a medium doping concentration combined with an intermediate n-layer thickness, about 100 nm, yields the best balance between breakdown voltage and specific on-resistance. However, excessive doping can also reduce breakdown voltage by narrowing the depletion region. In this regime, the current is injected mainly around the trench bottom corner, whereas the electric field is distributed along the p–n interface at the trench bottom, so an optimized trench-length ratio can further increase $V_{\mathrm{BD}}$ [135]. A separate study of a vertical PND structure with step-edge termination and a p–n-junction-based junction-termination extension predicted $R_{\mathrm{on},\mathrm{sp}}\approx 0.415$ mΩ·cm^2^, a breakdown voltage of approximately 2.2kV, and a Baliga figure of merit of $12.74\;\mathrm{GW}/{\mathrm{cm}}^{2}$ [136]. These results show that geometric field management can partly mitigate material-related limitations.

Recent reports show growing interest in heterojunction p–n devices and advanced field-termination designs, rather than only pure diamond homojunctions. These approaches can reduce the dependence on difficult n-type diamond doping while still using diamond’s high thermal conductivity and high breakdown field. For example, a kV-class vertical n–p rectifier based on an ITO/diamond heterojunction showed a breakdown voltage of about 1.1 kV and a specific on-resistance of about 13 mΩ·cm^2^. The reported device also had a power figure of merit close to 100 MW/cm^2^, an on-voltage of about 1.4 V, and a reverse-recovery time on the order of 10 ns for devices with a diameter of about 100 μm [138]. Diamond/ε-Ga_2_O_3_ ultrawide-bandgap heterojunction p–n diodes have also shown breakdown voltages above 3 kV without complex edge termination. In that case, the high breakdown voltage was linked to the sharp heterointerface and the lightly doped diamond drift layer, which helped reduce electric-field crowding [139].

Although these results are promising, the long-term reliability of diamond heterojunction diodes has not yet been established, since available studies mainly report initial electrical and breakdown characteristics. Extended bias-temperature stress, repetitive switching, and thermal-cycling data remain limited. Such testing is needed to determine whether interface degradation or thermomechanical stress will constrain practical operation.

Double-layer junction-termination extension (JTE) designs have also been proposed for diamond p–n diodes. Numerical studies suggest that stacked JTE regions can flatten the surface electric field and raise the breakdown voltage without causing a large increase in $R_{\mathrm{on},\mathrm{sp}}$ [181]. These results show that diamond p–n and p–i–n diodes have moved beyond simple homojunction structures. Reported devices now include sub-kV to >10-kV p–i–n diodes, trenched p–n and PND structures with improved field control, and newer heterojunction rectifiers for high-power and high-temperature operation. Even so, reproducible n-type doping remains a major barrier to broader use of diamond diode technology.

Overall, diamond diodes currently provide the strongest experimental evidence of high-voltage device capability, although reproducibility, junction control, and scalable fabrication remain unresolved. Diamond transistors face additional challenges associated with surface-channel stability, gate-stack reliability, and normally off operation, as discussed in the following section.

### 6.3. Diamond FETs

Diamond FETs have developed through several device concepts, including hydrogen-terminated surface-channel devices, MOS-gated structures, inversion-mode devices, deep-depletion MOSFETs, MESFETs, JFETs, and heterostructure FETs. Much of the early work focused on hydrogen-terminated surface-channel devices, whereas more recent studies have explored MOS-gated structures, inversion-mode operation, and heterostructure FETs. In general, diamond FETs are classified as surface-channel and bulk-channel types. Surface-channel FETs are based on hydrogen-terminated transfer doping to create a two-dimensional hole gas (2DHG). On the other hand, bulk-channel FETs operate through depletion or inversion in intentionally doped diamond layers [69]. Various types include MOSFETs, H-FETs, I-FETs, deep-depletion MOSFETs, Schottky-gated MESFETs, and JFETs [53,182,183,184].

However, each architecture exploits diamond’s large bandgap, high critical electric field, and high thermal conductivity through different device mechanisms. Certain architectures have primarily been focused on the development of devices capable of operating in the normally off mode, while other FET structures have stressed the high-temperature capability or high-field blocking ability. In this context, recent reports on normally off diamond MOSFETs suggest meaningful progress in threshold-voltage control through gate-stack and interface engineering [185,186,187]. Nevertheless, no single transistor architecture has yet emerged as clearly dominant. Threshold-voltage control, carrier mobility, manufacturing complexity, and reliability remain strongly coupled, making simultaneous optimization difficult.

Table 4 summarizes the main diamond FET architectures and their reported trade-offs. It highlights recent progress in normally off operation, vertical scaling, and gate-stack engineering for high-temperature and high-power applications.

#### 6.3.1. Metal–Oxide–Semiconductor Field-Effect Transistors (MOSFETs)

Metal–oxide–semiconductor field-effect transistors (MOSFETs) continue to be among the prominent transistor architectures explored in diamond-based electronics. This interest is mainly due to the gate stack, which provides enhanced electrostatic control and, in certain design schemes, allows normally off operation that can be particularly useful in power devices. However, MOSFET development in diamond has remained challenging. Many reported devices are still affected by surface-charge instability, high trap density at the interface, and poor control of either the depletion or the inversion channel. The presence of a wide bandgap in diamond poses an additional problem; minority-carrier generation required for conventional inversion-channel operation is strongly limited. Surface termination, interface quality, and dielectric processing therefore become central to device performance. Early diamond MOSFETs were fabricated using diamond epilayers with SiO_2_ gate dielectrics [192]. Later studies revealed hydrogen-terminated MOSFETs employing surface-channel conduction [193] and inversion-mode transistors fabricated on boron-doped oxygen-terminated diamond [194].

MOSFETs based on diamond are generally categorized into hydrogen-terminated and oxygen-terminated platforms. In hydrogen-terminated devices, a two-dimensional hole gas forms near the surface through surface transfer doping. In oxygen-terminated devices, this surface channel is eliminated, and depletion or inversion effects in doped diamond layers are emphasized. This means that these two kinds of platforms have different characteristics of threshold-voltage control and interface reliability [69].

**Figure 5:**
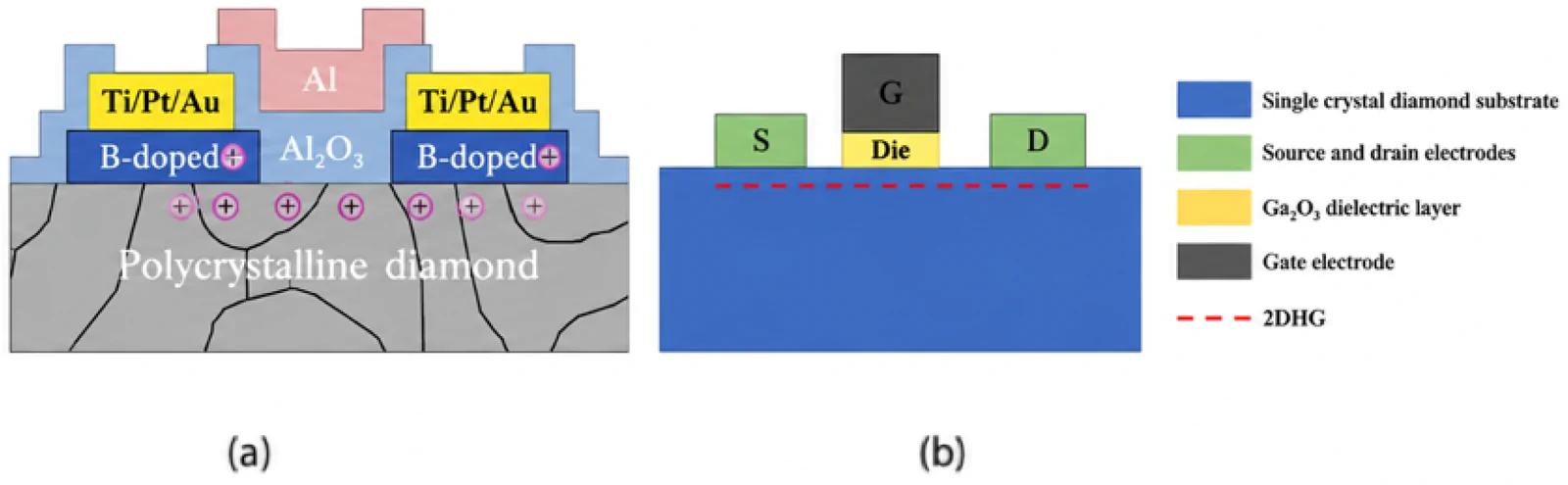
Evolution of hydrogen-terminated diamond MOSFET architectures based on surface two-dimensional hole gas (2DHG) channels. (**a**) Hydrogen-terminated MOSFET with engineered gate dielectric and electrostatic source/drain design to improve gate control and suppress leakage while retaining surface-channel transport [185]. (**b**) Normally off H-terminated diamond MOSFET using a Ga_2_O_3_ gate dielectric, enabling enhancement-mode operation through interface and gate-stack engineering [187]. Adapted from [185,187].

Normally off behavior has recently been demonstrated in hydrogen-terminated and boron-doped oxygen-terminated diamond MOSFETs [185,186,187]. These demonstrations appear to rely mainly on gate-stack optimization, interface management, and device scaling (Figure 5a,b). Nevertheless, integrating stable normally off operation, high mobility, low leakage, and reliable interfaces in a single diamond MOSFET remains challenging.

#### 6.3.2. Hydrogen-Terminated Diamond-Based MOSFETs

“Hydrogen-terminated diamond” refers to a diamond surface terminated by carbon–hydrogen bonds. These bonds produce a surface dipole and can lead to negative electron affinity (NEA). Under suitable conditions, electrons may transfer from the diamond valence band to acceptor states near the surface, such as adsorbed molecules with high electron affinity or oxide-related surface states. The holes left behind then form a high-density two-dimensional hole gas (2DHG), which is usually confined to only a few nanometers below the surface.

This surface-induced hole channel is the basis for accumulation-mode p-channel MOSFETs in hydrogen-terminated diamond. It allows conduction without intentional bulk doping in the channel. The channel density and stability, however, depend strongly on the surface environment. Nitrogen- or oxygen-containing adsorbates, for example, may change the type and density of acceptor states near the surface. In that sense, the behavior is not controlled by hydrogen termination alone but by the surface chemistry around it [195,196]. Because the channel depends on adsorbed or surface-related acceptor states, environmental stability and passivation remain important issues for H-terminated devices.

High sheet hole concentrations, reaching the 10^13^–10^14^ cm^−2^ range, have been reported after controlled exposure to strong electron-accepting species such as NO_2_ [195,196]. A wide range of materials and surface conditions have been reported to enhance hole accumulation on hydrogen-terminated diamond, including ambient air, NO_2_, V_2_O_5_, Al_2_O_3_, MoO_3_, AlN, fullerenes, and fluorinated fullerene derivatives. Detailed studies have shown that the increase in hole concentration observed under ambient conditions is primarily associated with adsorbed species such as NO_2_, O_3_, SO_2_, and NO, while molecules such as H_2_O, CO_2_, and N_2_O have minimal influence on hole carrier generation [73,74]. Although NO_2_-adsorbed hydrogen-terminated diamond MOSFETs can exhibit very high current densities, the induced hole channel is often unstable at elevated temperatures due to desorption of surface species. To address this limitation, atomic layer deposition (ALD) has been widely employed to deposit thin dielectric layers, such as Al_2_O_3_, which act as p-channel passivation layers while preserving the underlying C–H termination. Using this approach, Al_2_O_3_/NO_2_/H-diamond FETs have been demonstrated to operate stably at temperatures up to 400 °C with current densities as high as 1.35 A mm^−1^, highlighting the importance of dielectric encapsulation for thermal stability. These results show that channel performance is strongly linked to surface chemical control rather than bulk doping alone.

To further improve electrostatic gate control and suppress gate leakage, numerous gate oxides have been investigated for hydrogen-terminated diamond MOSFETs, including HfO_2_ [76], ZrO_2_ [197], Ta_2_O_5_ [198], LaAlO_3_ [199], and Y_2_O_3_ [200]. These materials are characterized by relatively high dielectric constants and serve two primary purposes: enhancing drain current density at reduced gate bias and improving gate insulation to limit leakage current. Experimental studies have consistently shown that bilayer or multilayer dielectric stacks deposited on hydrogen-terminated diamond surfaces are more effective at suppressing leakage and stabilizing device operation than single-layer oxides [201,202,203,204,205]. In parallel, significant effort has been devoted to improving the breakdown voltage of hydrogen-terminated diamond MOSFETs [77,78]. Strategies include increasing the gate–drain spacing, optimizing device geometry, and mitigating the effects of grain boundaries and surface defects in polycrystalline diamond. For example, a C–H-bonded channel device fabricated on black polycrystalline diamond, with a gate–drain length up to 20 μm, reached a breakdown voltage of about 1.8 kV and a current density of 1.1 mA mm^−1^. Hydrogen-terminated diamond MOSFETs with partially oxidized, or partial C–O, channels have also shown breakdown voltages above 2 kV at room temperature [206,207]. The current density in these devices is still relatively low, so further improvement is needed. These results show the familiar trade-off in diamond FETs: high blocking voltage is possible, but strong channel conduction is harder to maintain at the same time.

Recent studies have increasingly focused on normally off operation and interface control in hydrogen-terminated diamond MOSFETs. One example is a self-aligned Zr/ZrO_2_ gate structure, which reduced leakage current and produced a clearer enhancement-mode threshold voltage [208]. Other studies used optimized ZrO_2_/Al_2_O_3_ bilayer gate stacks deposited by electron-beam evaporation. These devices showed improved field-effect mobility and lower interface trap density compared with single-layer Al_2_O_3_ dielectrics [209]. Transfer-based h-BN gate dielectrics have also been tested, with reported improvements in channel mobility and stable gate modulation [210]. More recently, single-crystal hydrogen-terminated diamond MOSFETs using Ga_2_O_3_ as the gate dielectric showed normally off behavior, with on-state current magnitudes exceeding 30mA/mm while maintaining a positive threshold voltage [187] (Figure 5b).

Taken together, these reports suggest that hydrogen-terminated diamond MOSFETs are moving beyond surface chemistry alone. Gate-stack design and interface quality now play a major role in achieving stable enhancement-mode behavior. Even with this progress, long-term threshold-voltage stability and reproducible large-area processing still need further work before these devices can be used more broadly. Among the reported strategies, planar hydrogen-terminated MOSFETs based on gate-dielectric and interface engineering currently appear most compatible with scalable fabrication because they preserve the high-density surface channel and avoid the need for n-type diamond. Deep-depletion, FinFET, and inversion-mode structures can provide a more inherent normally off state, but presently involve lower drive current, more complex processing, or stricter interface-quality requirements. Nevertheless, long-term threshold stability and dielectric reliability must still be demonstrated before any approach can be considered commercially mature.

#### 6.3.3. Oxygen-Terminated Diamond-Based MOSFETs

Oxygen-terminated diamond refers to diamond surfaces terminated with C–O or C–OH bonds, where oxygen termination suppresses the surface transfer doping mechanism observed in hydrogen-terminated diamond and converts the surface from negative to positive electron affinity, resulting in an insulating surface. Therefore, the use of oxygen-terminated diamond in electronic devices is typically accompanied by intentional bulk doping and oxide-gated channel control. As a result, gate bias must induce conduction through bulk depletion or inversion mechanisms rather than through a surface-transfer-doped channel. In a notable study by Pham et al. [211], a comprehensive electrical analysis was conducted on metal/Al_2_O_3_/oxygen-terminated diamond capacitance structures. Building on this work, a deep-depletion MOSFET was fabricated by depositing Al_2_O_3_ onto a p-type boron-doped (100) oxygen-terminated diamond substrate at 380 °C using atomic layer deposition (ALD) [52]. This device demonstrated an exceptionally high hole mobility of 1000 ± 200 cm^2^ V^−1^ s^−1^, which was the highest reported value for oxygen-terminated diamond MOSFETs at the time, and achieved a breakdown voltage of approximately 200 V [51]. However, the corresponding on-state current density remained relatively low, approximately 1.9 μA mm^−1^, substantially smaller than that of hydrogen-terminated diamond MOSFETs. Detailed analysis revealed strong Fermi-level pinning at the Al_2_O_3_/diamond interface, arising from a combination of oxide leakage and a high density of interface states, estimated to be on the order of 10^12^ eV^−1^ cm^−2^. To address these limitations, the authors proposed a deep-depletion operating concept for diamond MOSFETs employing high-temperature ALD Al_2_O_3_ gate dielectrics [52]. In the deep-depletion regime, the ultrawide bandgap of diamond leads to a prolonged absence of inversion due to the extremely low intrinsic carrier concentration, particularly when no external source of minority carriers is available. These results illustrate that O-terminated devices may offer superior blocking behavior, but often at the expense of channel conductivity.

Inversion layers, which serve as conductive channels in conventional silicon MOSFETs, are therefore difficult to establish in diamond-based MOSFETs. The formation of inversion channels in diamond is particularly challenging because the intrinsic carrier concentration is extremely low, making the generation of minority carriers very slow. Kovi et al. [194] reported inversion behavior in boron-doped diamond MOS structures with a doping concentration of 4.1 × 10^19^ cm^−3^; however, such heavy doping resulted in substantial leakage currents and degraded electrical performance, limiting the practical usefulness of this approach. Subsequent work by Matsumoto et al. [53] demonstrated p-channel planar MOSFETs incorporating a phosphorus-doped n-type body on diamond (111) substrates using selective microwave plasma chemical vapor deposition, and later directly observed inversion capacitance in p-type diamond MOS capacitors. Despite this progress, these devices continued to suffer from very low drain current density and device cycling instabilities. Band-alignment analysis has proven essential for better understanding carrier transport in oxygen-terminated diamond MOS structures. Maréchal et al. [212] employed X-ray photoelectron spectroscopy (XPS) to establish the interfacial energy band diagram of Al_2_O_3_ deposited by ALD at 250 °C on oxygen-terminated boron-doped (001) diamond. The resulting band alignment was identified as Type I, with a valence-band offset $\Delta E_{v}=1.34\pm 0.20$ eV and a conduction-band offset $\Delta E_{c}=0.56\pm 0.20$ eV, assuming an Al_2_O_3_ bandgap of 7.4 eV. By comparison, Al_2_O_3_ deposited on hydrogen-terminated diamond shows a Type-II alignment and a much larger valence-band offset, $\Delta E_{v}\approx 2.90\pm 0.20$ eV, which points to the strong role of surface termination in shaping the interfacial electronic structure. This difference makes it clear that surface chemistry can strongly influence gate-stack behavior and, in turn, the operating mode of diamond MOSFETs.

For oxygen-terminated diamond MOSFETs, recent work has placed less emphasis on maximum current density and more on normally off operation, high breakdown voltage, and interface stability. Liu et al. reported normally off boron-doped diamond MOSFETs on oxygen-terminated surfaces with breakdown voltages above 1.7 kV. This result suggests that oxide-gated O-terminated structures can provide kilovolt-class blocking while still maintaining enhancement-mode operation [186]. Ota et al. also demonstrated vertical diamond MOSFETs using an oxidized Si-terminated diamond channel. In this structure, controlled surface oxidation helped stabilize the depletion channel and enabled normally off behavior, with drain current densities above 100 mA mm^−1^ and breakdown fields above 3 MV cm^−1^ [189].

Recent studies on ALD-grown Al_2_O_3_ interfaces have also shown progress in reducing gate leakage and limiting Fermi-level pinning on oxygen-terminated diamond. These improvements support the use of O-terminated diamond MOSFETs in high-temperature and high-voltage applications where threshold-voltage stability is particularly important. Even so, interface trap densities at the oxide/diamond interface remain a major obstacle to stable threshold control and strong inversion-channel formation. Further improvements in dielectric deposition, interface passivation, and defect control will likely be needed before high-performance inversion-mode diamond MOSFETs can be fully realized. Overall, oxygen-terminated MOSFETs appear especially promising for normally off and high-voltage applications, provided that their current-drive limitations can be reduced further.

#### 6.3.4. Deep-Depletion Diamond (D^3^MOSFET) and Inversion-Mode MOSFETs

Diamond’s ultrawide bandgap, 5.47 eV at room temperature, gives it several advantageous properties for electronic devices, particularly MOSFETs. Most notably, the extremely low intrinsic carrier concentration strongly suppresses thermally generated minority carriers, which plays a central role in enabling and sustaining deep-depletion operation [213]. Consequently, the generation of minority carriers required to form a conventional inversion layer is extremely slow. In contrast to conventional semiconductors such as silicon, where deep depletion is a transient, non-equilibrium effect that depends sensitively on time, temperature, and bias sweep rate, diamond exhibits a unique deep-depletion behavior that can remain stable over extended timescales and wide temperature ranges [213,214,215]. In silicon MOSFETs, deep depletion typically enhances only the dynamic breakdown voltage and collapses once minority carriers are generated, whereas in diamond, the delayed onset of inversion is an intrinsic consequence of its wide bandgap and low intrinsic carrier density. As a result, the device can remain in a deep-depletion state over a wide range of gate biases without forming a conventional inversion layer, behaving more like a depletion-mode transistor than a classical inversion-mode MOSFET. Since conduction occurs through the bulk rather than through a high-mobility surface channel, the achievable current density is generally lower than in surface-channel H-terminated FETs. This highlights the central trade-off of deep-depletion devices: improved blocking behavior at the expense of channel drive current.

Experimental deep-depletion diamond MOSFETs have been demonstrated using Al_2_O_3_/(Ti/Pt/Au) gate stacks, achieving high breakdown electric fields approaching 4 MV cm^−1^ in lateral normally on device configurations [51]. Despite these encouraging breakdown characteristics, the maximum drain current density observed in such devices remains significantly lower than that of hydrogen-terminated diamond FETs, reflecting the absence of a high-density surface hole channel. A comprehensive investigation of deep-depletion diamond MOSFETs, commonly referred to as D^3^MOSFETs, further confirmed their feasibility and stable transistor operation [51]. These devices exhibited conventional MOSFET characteristics, with a specific on-state resistance as low as 50 mΩ·cm^2^ at 250 °C and a maximum breakdown voltage of approximately 175 V [216]. Breakdown was found to occur primarily within the gate oxide due to electric-field crowding, highlighting the importance of improved oxide robustness and edge-termination design. A key advantage of D^3^MOSFETs is diamond’s exceptionally high thermal conductivity, which facilitates efficient heat spreading during high-power operation. However, the observed negative temperature coefficient of the drain current requires careful consideration when paralleling devices to ensure stable current sharing. Overall, these results indicate that while D^3^MOSFETs exhibit promising thermal and breakdown properties, further optimization of device geometry, gate dielectrics, and termination schemes is required to fully exploit their performance potential [217].

**Figure 6:**
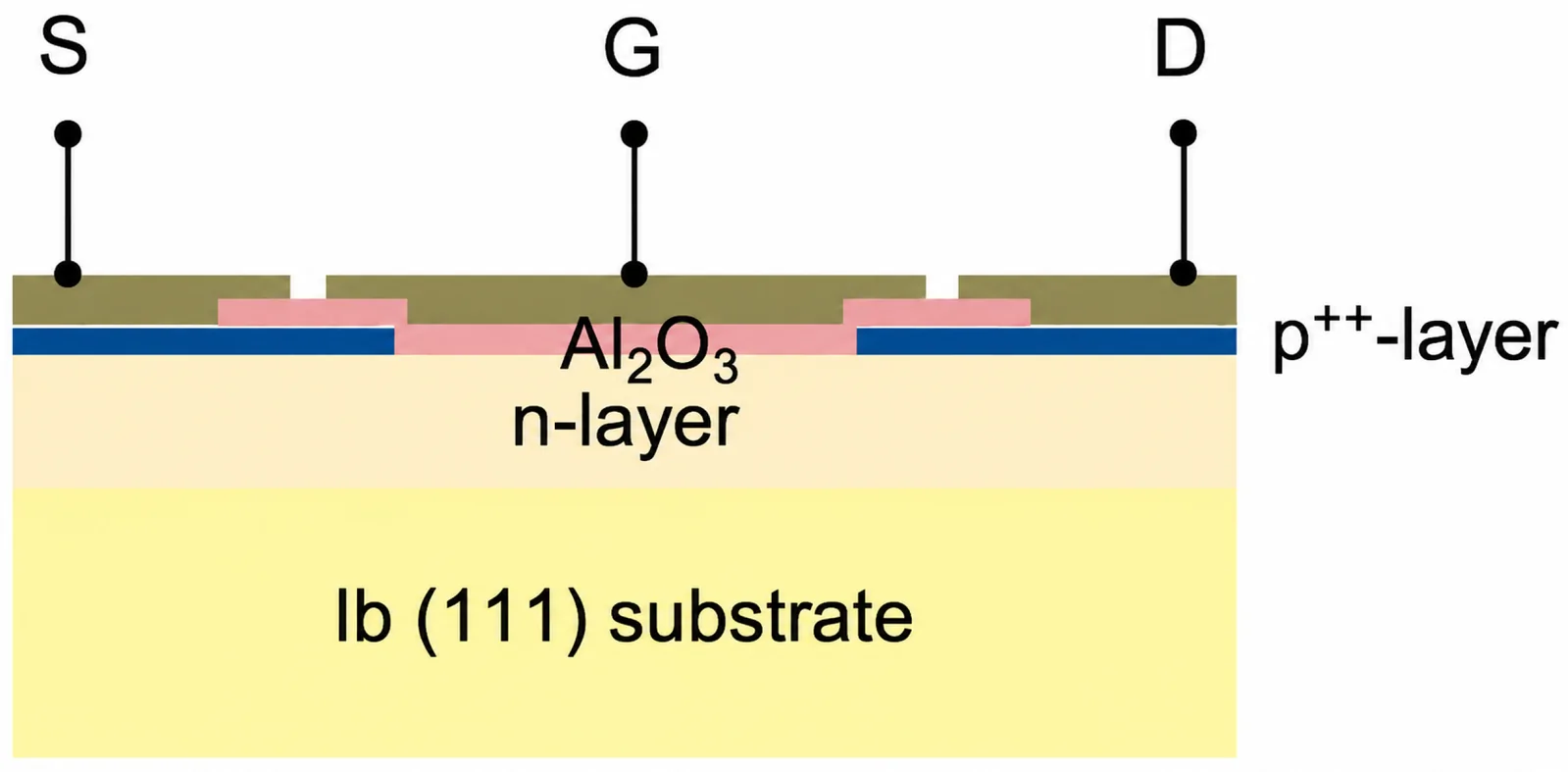
Inversion-channel diamond MOSFET structure enabling normally off behavior through electrostatically induced channel formation [53].

Recent advances have extended deep-depletion concepts to more advanced device geometries. Deep-depletion diamond FinFETs have been demonstrated using chemical vapor deposition (CVD) boron doping on (100)-oriented, 3 × 3 mm_2_ HPHT undoped substrates. In these devices, fin structures were fabricated by electron-beam lithography and O_2_ dry etching. A relatively low boron concentration in the channel, 5 × 10^16^ cm^−3^, combined with a 45 nm thick SiO_2_ gate oxide and a low metal work-function aluminum gate, approximately 4.08 eV, resulted in a depletion width of approximately 55 nm. Because this depletion width exceeded half of the fin channel width, normally off operation was achieved. In parallel, high-quality p-doped diamond body/Al_2_O_3_ interfaces, realized through wet annealing, enabled the first demonstration of an inversion-type lateral diamond MOSFET on a (111) HPHT substrate, as reported by Matsumoto et al. [53] (Figure 6). That device exhibited a maximum drain current density of 1.6 mA mm^−1^ and a channel field-effect mobility of 8 cm^2^ V^−1^ s^−1^ at $V_{\mathrm{GS}}=-12$ V and $V_{D}=-5$ V. Although normally off operation was achieved, the relatively large magnitude of the threshold voltage, $V_{\mathrm{th}}\approx -6.3$ V, indicated a high density of interface traps, estimated to exceed 6 × 10^12^ cm^−2^ eV^−1^ [53]. These findings reinforce that interface quality remains a dominant limiter even when channel design is improved. In this device, the combination of $D_{\mathrm{it}}>6\times 10^{12}\;{\mathrm{cm}}^{-2}\;{\mathrm{eV}}^{-1}$, a field-effect mobility of only $8\;{\mathrm{cm}}^{2}\;V^{-1}\;s^{-1}$, and $V_{\mathrm{th}}\approx -6.3\;V$ illustrates how interface trapping can simultaneously weaken carrier transport and complicate threshold-voltage control. Direct numerical comparison among studies should nevertheless be made cautiously because the extracted $D_{\mathrm{it}}$ depends on the measurement method, assumed energy distribution, and gate-stack structure.

More recent studies have revisited deep-depletion diamond MOSFETs with a focus on achieving robust normally off operation, improved high-temperature stability, and scalability toward higher blocking voltages. Liu et al. reported normally off boron-doped diamond MOSFETs operating in a deep-depletion regime with breakdown voltages exceeding 1.7 kV, demonstrating that depletion-controlled bulk channels can support kilovolt-class blocking without relying on surface transfer doping [186]. In a complementary vertical device approach, Ota et al. demonstrated normally off diamond MOSFETs employing an oxidized Si-terminated diamond channel, in which controlled surface oxidation stabilized deep-depletion behavior and enabled drain current densities above 100 mA mm^−1^ together with breakdown fields exceeding 3 MV cm^−1^ [189]. These results indicate that deep-depletion concepts, once regarded primarily as an academic curiosity, are now emerging as viable and scalable design strategies for normally off diamond power MOSFETs capable of operation at high temperature and high electric field. If current-density limitations continue to improve, this architecture could become one of the most practical routes toward normally off diamond power transistors.

#### 6.3.5. Metal–Semiconductor FETs, Junction FETs, and Bipolar Junction Transistors

Beyond MOS-based architectures, metal–semiconductor field-effect transistors (MESFETs) and p–n junction field-effect transistors (p–n JFETs) offer high reliability for power and harsh-environment electronics, primarily due to the absence of a gate oxide layer. In conventional MOSFETs, the gate dielectric and its interface with the semiconductor can introduce high densities of interface states and trapping/de-trapping phenomena that degrade performance, particularly with high electric field, high temperature, or radiation exposure. Oxide-free device concepts therefore provide a more robust alternative for diamond electronics. Umezawa et al. investigated diamond MESFETs employing different Schottky gate materials, including molybdenum (Mo), platinum (Pt), and aluminum (Al) [183].

These devices exhibited a maximum drain current density of approximately 1.2 mA mm^−1^ at elevated temperatures, around 600 K, with zero gate-source bias ($V_{\mathrm{GS}}=0$ V) and a drain-source voltage of −20 V, with the enhanced conduction attributed to thermally activated boron acceptors in the channel region. The same devices demonstrated high breakdown voltages exceeding 2 kV for gate-to-drain spacings of 50 μm [218]. Diamond MESFETs typically operate in a normally on mode with relatively large threshold voltages, and their breakdown voltage scales favorably with gate–drain distance [183]. In addition to conventional lateral designs, diamond MESFETs have also been implemented in reverse-blocking configurations using Schottky metal contacts for both the gate and drain, further expanding their applicability in power switching circuits [218]. These features make MESFETs attractive for applications where thermal and gate-stack robustness are prioritized over maximum transconductance or aggressive scaling.

In parallel, advances in the growth and control of n-type diamond layers, particularly along the 〈111〉 crystallographic direction, have enabled the fabrication of diamond p–n^+^ junctions exhibiting high rectification ratios and breakdown voltages approaching 1 kV [184,219,220,221]. These p–n^+^ junctions have been employed as the fundamental building blocks of diamond JFETs [222]. By varying channel width and doping concentration, diamond JFETs exhibiting both normally on and normally off characteristics have been demonstrated, supporting operation in either unipolar or bipolar conduction modes. The breakdown voltage of these devices shows a positive temperature coefficient, attributed to increased phonon scattering and the resulting reduction in avalanche multiplication at elevated temperatures [184]. In particular, normally off JFETs with threshold voltages around −1.2 V have been realized by simultaneously reducing the channel doping concentration and channel width, thereby fully depleting the channel at zero bias. While such devices maintain high rectification ratios, they typically exhibit reduced current density due to the increased resistivity of the lightly doped channel region [191]. At elevated temperatures, however, a positive shift in the threshold voltage can drive these devices back into normally on operation, a behavior that has been confirmed through both TCAD simulations and experimental measurements [190,223]. Furthermore, operation in bipolar mode, where minority carriers, electrons, are injected into the p-type channel, has been shown to enhance current density in both normally on and normally off JFETs. This improvement comes at the expense of slower turn-off dynamics and increased gate-drive complexity due to stored charge effects. Thus, JFET designs often trade switching simplicity for improved current capability.

Recent progress in n-type doping technology has also enabled the fabrication of diamond bipolar junction transistors (BJTs) [224,225]. Early attempts at diamond BJTs were limited by the high resistivity of the n-type base region, typically on the order of 10^18^ cm^−3^, and the short diffusion length of minority carriers, holes, within the base. The use of heavily doped n^+^ layers has helped improve device performance by increasing hopping conductivity and lowering series resistance through low-resistance ohmic contacts to the base region. Even with these improvements, diamond BJTs are still difficult to scale, mainly because of the short minority-carrier diffusion length and the challenge of controlling the base width with sufficient precision [225]. For this reason, BJTs currently remain more specialized than the more widely studied diamond FET concepts.

In recent years, junction- and Schottky-gated diamond transistors have attracted renewed attention, particularly for applications where thermal stability and radiation tolerance are more important than conventional MOS gate control. Okuno et al. reported normally off diamond MESFETs intended for digital circuit operation and tested their behavior under high-temperature and radiation-rich conditions. Their results showed stable switching in environments where oxide-gated devices may face reliability concerns [226]. At the same time, progress in selective doping and device layout has improved the reproducibility and gate control of diamond JFETs. Recent reports have shown lower leakage currents and better electrostatic control by optimizing the junction profile and device geometry [184,191]. Although diamond BJTs still fall behind FET-based devices in current gain and switching speed, continued work on n-type doping and contact engineering keeps bipolar diamond devices relevant for certain high-power and extreme-environment applications. Overall, oxide-free transistor designs remain an important complementary route, especially in cases where robustness and environmental tolerance matter more than the advantages offered by standard MOS gate structures.

#### 6.3.6. Two-Dimensional Hole Gas-Based (2DHG) Field-Effect Transistors

**Figure 7:**
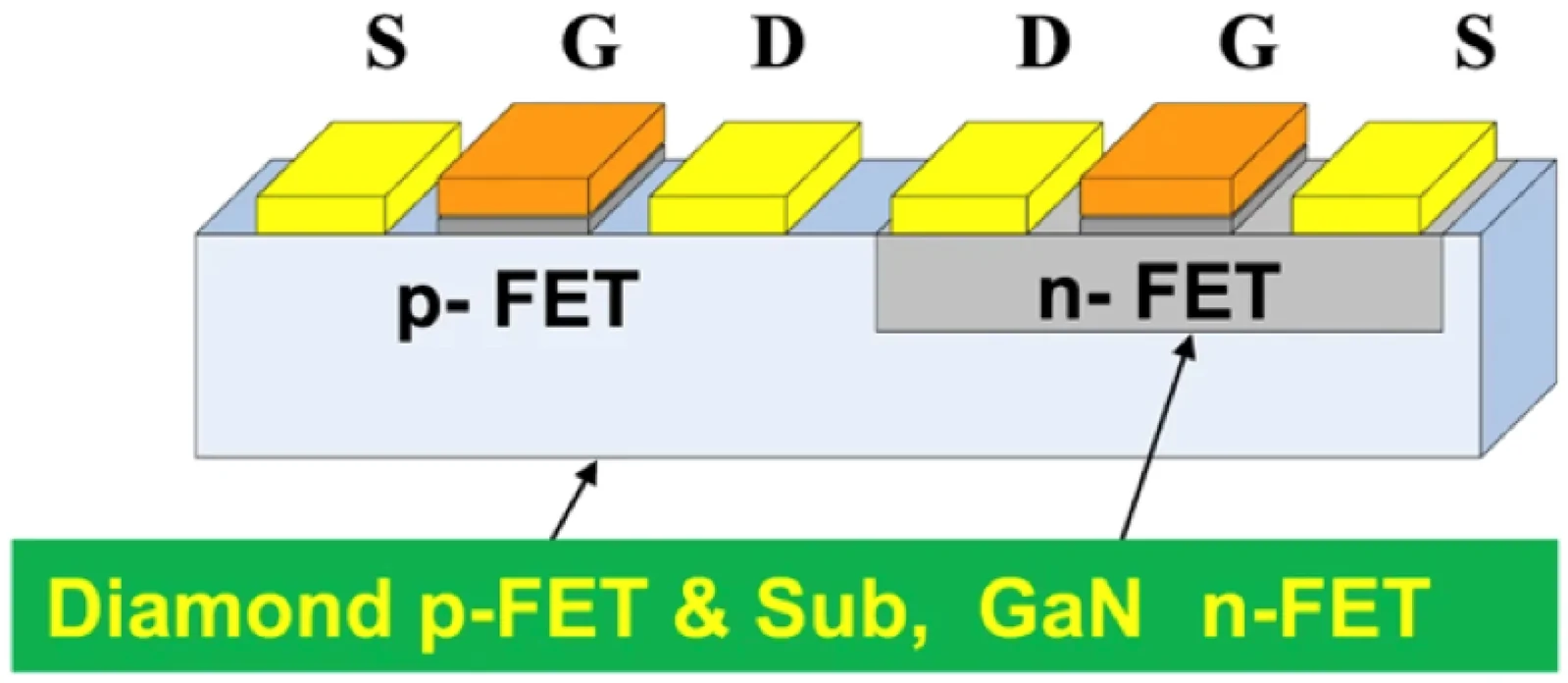
Durability-enhanced 2DHG device formed on a hydrogen-terminated diamond (C–H) surface, demonstrating stable surface transfer doping suitable for complementary power inverter applications [70].

Two-dimensional hole gas (2DHG)-based field-effect transistors exploit a unique transport mechanism arising from the formation of a high-density hole channel near hydrogen-terminated diamond surfaces. This concept, first identified in the early 1990s, enables the creation of a surface-confined hole gas with nearly zero activation energy, making it possible to realize surface-channel FETs without intentional bulk doping [227,228,229,230,231]. Owing to this mechanism, 2DHG-based diamond FETs have attracted significant interest as candidates for high-temperature, RF, and selected power-electronic applications [182]. Typical devices exhibit peak channel mobilities on the order of 100 cm^2^ V^−1^ s^−1^ and sheet hole densities ranging from 10^12^ to 10^14^ cm^−2^, particularly when strong surface acceptors such as NO_2_ are employed to enhance surface transfer doping [232,233]. To improve the reliability and long-term stability of heterojunction field-effect transistors (HFETs), atomic layer deposition (ALD) of Al_2_O_3_ has been widely used to uniformly induce and stabilize hole accumulation at the interface [233,234]. In these structures, unoccupied states or fixed charges associated with the surface/insulating layer have been proposed to help sustain the 2DHG at the diamond surface [70] (Figure 7). Direct comparison of reported 2DHG FET performance also requires caution because mobility and current density depend on surface-acceptor chemistry, dielectric-stack design, contact resistance, device dimensions, bias conditions, and the extraction method. Moreover, device-to-device statistics and extended bias-temperature stability data remain unavailable in many reports.

A wide range of lateral HFET architectures based on 2DHG channels has been demonstrated in the literature, including normally on devices exhibiting breakdown voltages exceeding 1.5 kV and stable operation at temperatures above 725 K [76,201,235,236,237,238]. Compared with conventional lateral HFETs, lateral triple-gate HFETs, which permit carrier transport in both lateral and vertical directions, have shown enhanced current density and offer more favorable scaling prospects for device miniaturization [239]. Despite these advantages, most 2DHG-based HFETs remain normally on due to the intrinsic nature of surface transfer doping. Consequently, considerable effort has been devoted to suppressing the formation of the 2DHG under the gate region in order to achieve enhancement-mode operation. Strategies including the use of double high-*k* dielectric gate stacks have been explored to suppress surface acceptor states and reduce hole accumulation beneath the gate [76,201,235,236,237,239,240], as well as partial oxidation of the channel region to locally convert C–H bonds into C–O bonds, thereby enabling normally off behavior [77]. This persistent normally on tendency remains one of the main barriers to power-switching adoption.

Unlike AlGaN/GaN heterostructures, where two-dimensional carrier gases arise primarily from piezoelectric polarization effects, 2DHG formation in diamond is governed by surface chemistry and electronic structure. As a result, 2DHG channels in diamond can be realized in non-planar geometries, including vertical trench structures, offering additional flexibility in device design [241,242]. However, residual bulk doping and incomplete electrical isolation can lead to temperature-dependent leakage currents in HFETs, particularly at elevated operating temperatures. To address this issue, in situ annealing of hydrogen-terminated diamond surfaces prior to oxide deposition has been shown to significantly enhance long-term doping stability and reduce degradation during high-temperature operation [241]. These advances suggest that hydrogen-terminated diamond HFETs remain promising candidates for extreme-environment electronics and may become suitable for specialized high-temperature applications if manufacturability and threshold stability are further validated.

Recent studies on 2DHG-based diamond FETs have increasingly emphasized consolidation of device-physics understanding and assessment of realistic performance limits, rather than the introduction of fundamentally new device architectures. A comprehensive review by Kawarada summarizes progress in hydrogen-terminated diamond p-channel FETs employing 2DHG channels, highlighting steady improvements in surface stabilization, gate dielectric integration, and radio-frequency (RF) device metrics such as drain current density and cutoff frequency [21]. While drain current densities exceeding 1 A mm^−1^ and encouraging high-frequency performance have been reported, the review underscores that robust normally off operation remains a central unresolved challenge for 2DHG-based devices. Consequently, current research trends increasingly position 2DHG diamond FETs as attractive candidates for high-frequency, analog, and complementary circuit applications, while recognizing that MOSFET-based architectures are better suited for power switching roles requiring strict enhancement-mode operation. Collectively, these observations indicate that 2DHG-based diamond FETs have advanced to the point where stability, reproducibility, and integration are now as important as further architectural innovation.

### 6.4. Emerging Diamond Device Platforms: MEMSs/NEMSs, High-Frequency Resonant Devices, and Memory Concepts

In addition to diamond rectifiers and diamond FETs, diamond is also being studied for a number of emerging device platforms that take advantage of the material’s mechanical rigidity, thermal stability, wide bandgap, radiation hardness, and defect-related charge dynamics. While such technologies may be somewhat less mature than diamond rectifiers and FETs, they have been included in this review because they rely heavily on many of the same materials and processes discussed throughout the previous sections. These include diamond MEMSs/NEMSs, high-frequency resonant devices, and memory concepts.

#### 6.4.1. Diamond MEMSs/NEMSs and High-Frequency Resonant Devices

Microelectromechanical systems (MEMSs) and nanoelectromechanical systems (NEMSs) have attracted substantial interest for a wide range of applications, including accelerometers, biosensors, and chemical sensors. Diamond has emerged as an attractive material platform for NEMSs/MEMSs owing to its exceptional physical, chemical, electrical, and tribomechanical properties, which enable stable operation under harsh mechanical, thermal, or chemical conditions [243,244,245,246,247,248]. Significant progress has been made in the development of diamond-based NEMS/MEMS structures using single-crystalline, polycrystalline, nanocrystalline, and ultrananocrystalline diamond films. These micro- and nanoscale mechanical resonators are particularly promising for ultrahigh-sensitivity sensing, precision metrology, and emerging quantum technologies.

In the context of quantum science and engineering, diamond mechanical resonators coupled to optical cavities or quantum spin systems have been extensively investigated. Tao et al. [249] demonstrated single-crystalline diamond cantilevers with thicknesses as low as 85 nm and lateral dimensions up to 240 μm, achieving ultrahigh mechanical quality factors exceeding 10^6^ at room temperature. These values surpass those of state-of-the-art single-crystalline silicon cantilevers of comparable size by nearly an order of magnitude. In parallel, diamond-integrated optomechanical circuits based on freestanding resonators and on-chip Mach–Zehnder interferometers achieved mechanical quality factors exceeding 11,200 [250]. Furthermore, active modulation of diamond nanophotonic circuits has been achieved by exploiting the mechanical flexibility of freestanding diamond electro-optomechanical resonators, which demonstrate quality factors above 9600 [251]. These results suggest that diamond can compete strongly with established MEMS materials in both mechanical and photonic domains.

**Figure 8:**
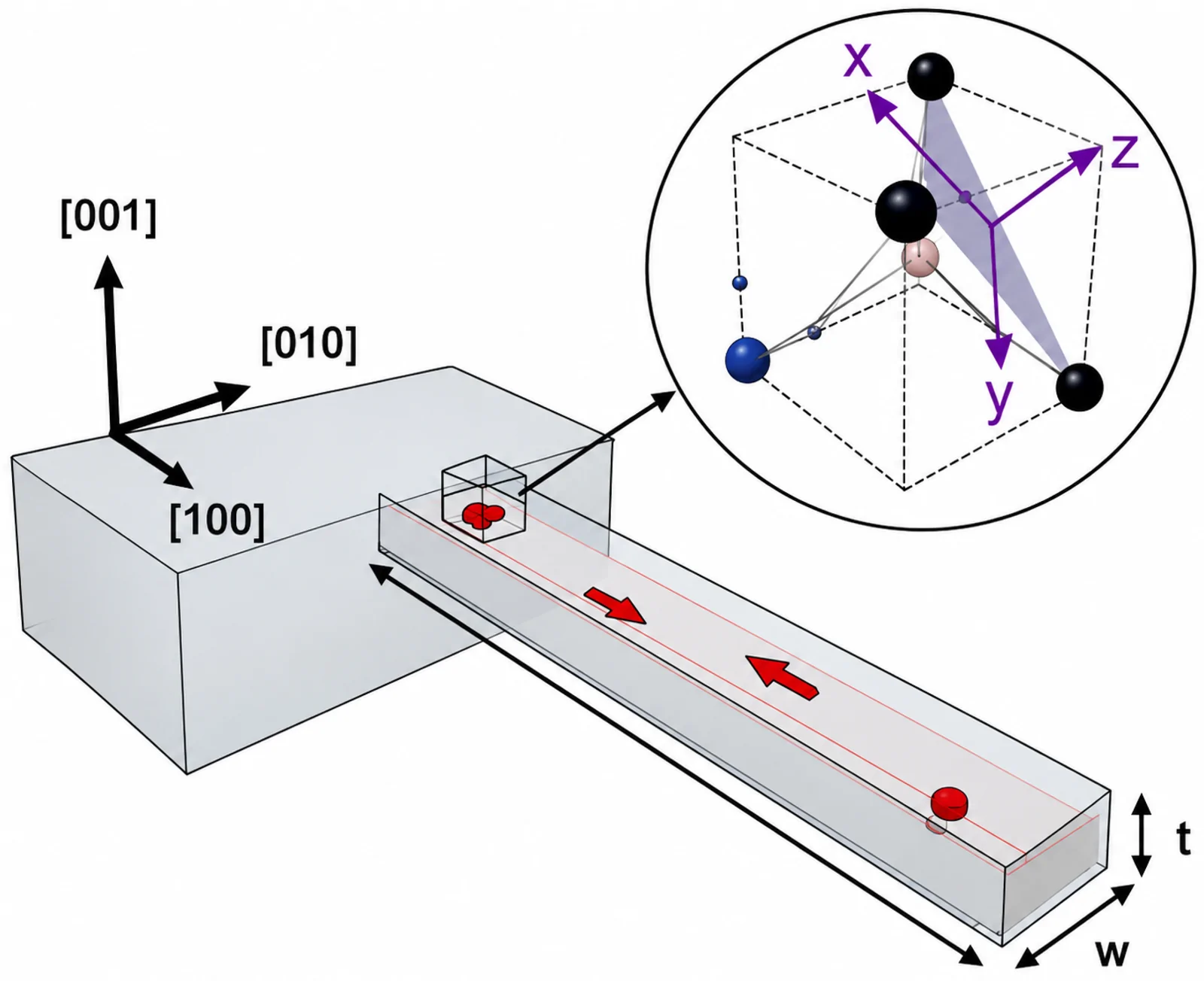
Strain-mediated coupling between a nitrogen–vacancy (NV) center spin and a diamond mechanical oscillator, illustrating coherent spin–mechanical interaction in diamond quantum NEMS platforms. The red arrows indicate NV-center spins, while the inset shows the color-coded atomic configuration and the *x*, *y*, and *z* coordinate axes [252].

Beyond classical optomechanics, diamond NEMS/MEMS platforms have enabled fundamental studies of hybrid quantum systems. Experimental work has shown strain coupling between nitrogen–vacancy (NV) center spins and diamond mechanical oscillators [252] (Figure 8). Related progress has also been made in diamond photonics. One- and two-dimensional photonic crystal microcavities have been fabricated in single-crystal diamond, with tunable coupling to color-center optical transitions and an emission-intensity enhancement of about 2.8 [120]. This kind of coupling between color centers and photonic crystal cavities is important for diamond-based photonic quantum systems, especially if the devices are to be integrated more broadly. More generally, the combination of mechanical, optical, and spin properties remains one of the reasons diamond is attractive for quantum and sensing technologies.

Recent work on diamond MEMSs and NEMSs appears to be moving less toward entirely new device concepts and more toward improving existing platforms. Much of the effort is now focused on higher mechanical quality factors, reduced surface loss, and fabrication processes that can be reproduced more reliably. Graziosi et al. demonstrated single-crystal diamond micro-disk resonators fabricated using deep reactive ion etching and focused ion beam milling, showing how controlled microfabrication can support compact diamond resonant structures for integrated photonic and optomechanical applications [253]. Kawarada also emphasized the role of diamond MEMSs/NEMSs in high-frequency and harsh-environment electronics, where thermal stability, mechanical strength, and long-term reliability are especially valuable [21].

These reports suggest that diamond MEMSs and NEMSs are becoming more technically mature, although the field still depends heavily on surface engineering, phononic and photonic integration, and scalable fabrication. Further improvements in diamond growth, microfabrication, and surface treatment should expand what these devices can do, but cost and large-area process scalability remain important barriers to broader use.

#### 6.4.2. Diamond-Based Memory Devices

Diamond-based memory devices represent one of the least explored yet most forward-looking directions in diamond electronics. Although substantially less mature than diamond diodes and field-effect transistors, they are important because diamond’s wide bandgap, deep defect states, thermal and chemical stability, and radiation tolerance may enable robust memory operation in harsh environments, particularly where long retention and reliability are more important than low cost or high storage density.

**Figure 9:**
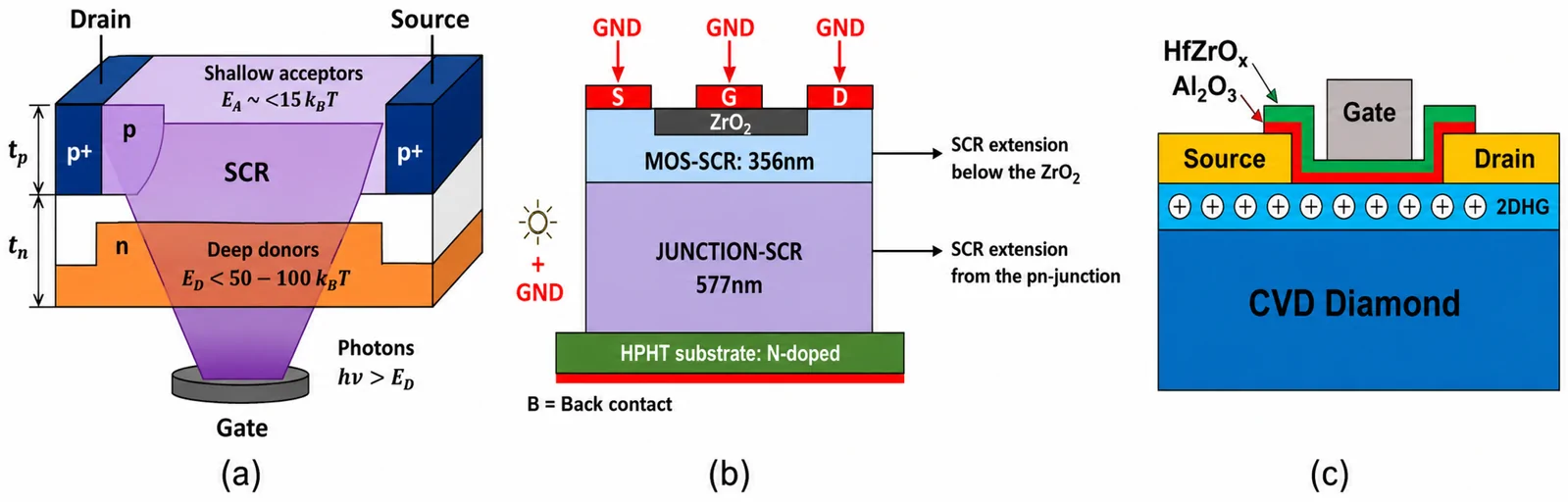
Representative diamond-based memory device concepts. (**a**) Diamond non-volatile photoswitch structure based on a p–n junction/JFET configuration, where optical excitation of deep donor states enables memory-like switching behavior [254]. (**b**) Diamond MOS memory-related structure showing depletion-region control and threshold-voltage modulation in a junction/MOS configuration [257]. (**c**) Ferroelectric-gate diamond MOSFET concept using an HfZrO_*x*_/Al_2_O_3_ gate stack on hydrogen-terminated diamond for memory-oriented operation [258]. Adapted from [254,257,258].

Diamond non-volatile photoswitches are one example. In these devices, a diamond p–n junction is used as the gate of a junction field-effect transistor (JFET). The memory response is linked to deep nitrogen donors in the n-type diamond region. Under illumination, these donors can be optically activated, allowing the gate to modulate the channel. When the light is removed, the written state may remain, giving a non-volatile response [254] (Figure 9a). Another work showed that the switching behavior depends on illumination wavelength, optical power density, and gate geometry [255]. Subsequent studies have examined ultra-deep nitrogen donors and suggest that their slow ionization dynamics could support long retention times. Reproducibility, doping control, and fabrication yield, however, remain significant problems for this approach [256].

Gate-stack engineering in diamond MOSFETs provides another possible memory route. Ferroelectric dielectrics, including HfZrO_*x*_/Al_2_O_3_ on hydrogen-terminated diamond, have produced hysteretic transfer behavior and a relatively wide memory window. This points toward diamond-based ferroelectric FET memory, or possibly negative-capacitance operation, although trap-related effects still need to be separated carefully from true ferroelectric behavior [258] (Figure 9c). In oxygen-terminated diamond MOS structures, memory-like threshold-voltage tuning has also been reported using deep-depletion behavior with ZrO_2_ gate dielectrics [257] (Figure 9b). This is closely connected to power-device operation because threshold shifts can affect whether a device behaves as normally on or normally off. Before these structures can be considered practical memory devices, several issues still need closer examination, including interface traps, gate leakage, polarization stability, retention, and endurance.

Diamond and diamond-related materials have also been explored for optical and resistive storage. In optical memory, data can be stored by controlling the charge state of color centers, particularly nitrogen-vacancy-related defects. Early studies showed that long-term information storage in diamond is possible through optical writing and reading of trapped charge states [259]. Later work pushed this concept toward higher-density optical storage using fluorescent vacancy centers [260]. Other carbon-based systems have also shown memory-related behavior, including multibit optoelectronic memory in graphene/diamond carbon sp^2^–sp^3^ heterojunctions and resistive switching in diamondoid thin films [261,262]. Although these examples are not conventional diamond MOS memories, they suggest that diamond and related carbon materials may support several forms of non-volatile storage.

At present, diamond memory technology remains much less developed than silicon flash, ferroelectric memory, or oxide-based resistive RAM. Its most realistic near-term use is probably not low-cost mass storage but rather specialized memory for high-temperature, radiation-rich, optoelectronic, or long-retention environments. Further progress will likely require cleaner oxide/diamond interfaces, improved doping control, stable surface termination, reduced programming energy, and more reproducible fabrication. If these challenges can be addressed, diamond memory devices may become a useful complement to diamond power electronics, especially for extreme-environment applications.

## 7. Conclusions and Future Perspectives

The reviewed literature shows that diamond has evolved from a conceptual ultrawide-bandgap semiconductor into a device platform under active development for power electronics and selected harsh-environment applications. Among the device classes discussed, diamond diodes appear to be the most mature, especially those operating with high voltage, high temperature, and high power. Diamond field-effect transistors have also made steady progress, particularly in hydrogen-terminated surface-channel and MOS-gated configurations. However, threshold-voltage control, normally off behavior, low contact resistance, interface stability, and reliability remain major challenges. Emerging platforms such as MEMSs/NEMSs, high-frequency resonators, UV photodetectors, and memory devices show that diamond applications are not limited to conventional switching devices. Even so, these areas are still at an earlier stage and remain limited by fabrication reproducibility, surface stability, and material quality.

Future progress should depend less on isolated high-performance demonstrations and more on resolving the practical issues that still limit reproducible diamond device operation. These include large-area and low-defect substrates, improved CVD growth, reliable dopant activation, reduced contact resistance, stable dielectric/diamond interfaces, surface-termination control, and scalable fabrication. Closing the gap between laboratory demonstrations and industrial deployment will require wafer-level evidence rather than isolated best-device results. Key milestones include uniform material and doping across larger substrates, repeatable fabrication processes with documented yield and device-to-device variation, and qualification with prolonged bias, thermal cycling, and repetitive switching. Until these requirements and manufacturable packaging are established, record breakdown or current-density values should be viewed primarily as demonstrations of feasibility rather than indicators of production readiness. These priorities can be considered across several development stages. In the near term, progress is most likely to come from improved contact resistance, dielectric-interface stability, surface control, and reproducible device processing. Over the medium term, larger low-defect substrates, more uniform CVD growth, stable normally off operation, and reliable high-temperature packaging will be required. Reproducible n-type doping, scalable bipolar-device fabrication, and cost-effective manufacturing remain longer-term challenges. These time frames are intended as approximate indicators of technological maturity rather than fixed predictions.

Over the next 5–10 years, the most realistic commercial opportunities for diamond devices are likely to remain in specialized, relatively low-volume applications where high temperature, radiation tolerance, or high-field operation can justify the added material and fabrication cost. These may include UV and radiation detectors, harsh-environment electronics, and selected pulsed-power or high-voltage protection switches. Broader adoption of diamond transistors in electric vehicles, industrial drives, and general grid converters is likely to require longer-term progress in wafer scalability, normally off operation, device reliability, packaging, and manufacturing cost. Diamond should therefore not be viewed simply as a direct replacement for SiC or GaN in established power-electronics markets. Its more realistic role is likely to be in specialized applications where high thermal conductivity, high breakdown strength, radiation tolerance, chemical stability, and surface-related electronic behavior provide a clear advantage.

## Figures and Tables

**Table 1:** Representative room-temperature electrical and thermal properties of Si, 4H-SiC, and GaN relevant to power and high-frequency semiconductor applications. The listed values are approximate and may vary with crystal orientation, material quality, doping concentration, and measurement method. Values summarized from [2,3,4,5].

Material	Semiconductor Class	Bandgap Energy (eV)	Critical Electric Field (MV cm^−1^)	Static Relative Permittivity $\varepsilon_{r}$	Intrinsic Carrier Concentration (cm^−3^)	Thermal Conductivity (W cm^−1^ K^−1^)	Electron Mobility (cm^2^ V^−1^ s^−1^)	Saturation Drift Velocity (cm s^−1^)
Si	Conventional	1.12	∼0.3	11.8	∼$1.08\times 10^{10}$	∼1.5	∼1300	∼$1.0\times 10^{7}$
4H-SiC	WBG	3.26	∼2.2–3.0	∼9.7	∼$10^{-8}$	∼3.7	∼900	∼$2.0\times 10^{7}$
GaN	WBG	3.4	∼3.3–3.5	∼9.5	∼$1.9\times 10^{-10}$	∼1.3	∼900–2000	∼$2.5\times 10^{7}$

**Table 2:** Representative material properties of selected wide-bandgap (WBG) and ultrawide-bandgap (UWBG) semiconductors. Adapted from [12].

Property	GaN	4H-SiC	AlGaN/AlN	*β*-Ga_2_O_3_	Diamond
Classification	WBG	WBG	UWBG	UWBG	UWBG
Bandgap (eV)	3.4	3.3	Up to 6.0	4.9	5.47
Thermal conductivity (W m^−1^ K^−1^)	253	370	253–319	10–27	2290–3450
Substrate defect density (cm^−2^)	∼10^4^	∼10^2^	∼10^4^	∼10^4^	∼10^5^
Substrate diameter (in.)	8 (on Si)	8	2	4	1
Demonstrated p-type dopability	Good	Good	Poor	No	Good
Demonstrated n-type dopability	Good	Good	Moderate	Good	Moderate

**Table 3:** Summary of reported diamond power diodes, including unipolar, bipolar, hybrid, and heterojunction architectures, key electrical parameters, and representative reported performance metrics.

Device Type	$V_{\mathrm{ON}}$ (V)	$R_{\mathrm{on},\mathrm{sp}}$	Reverse Current Density	Forward Current Density	Rectification Ratio	$E_{\mathrm{BR}}$ (MV/cm)	BV	Ref.
SPND	4	∼1 × 10^−5^ Ω·cm^2^	NR	>10^4^ A/cm^2^ @ ∼7 V	∼10^8^	NR	NR	[125]
MIPD	NR	4.15 × 10^−5^ Ω·cm^2^	2 nA/cm^2^ @ 10 V	7570 A/cm^2^ @ −10 V	≈10^12^ @ ±10 V	≈4.2	≈42 V	[126]
Schottky PiN	9.2	0.25 mΩ·cm^2^	NR	12.1 kA/cm^2^	NR	NR	NR	[127]
SBD	NR	1.31 mΩ·cm^2^	NR	4740 A/cm^2^	3.32 × 10^8^ @ ±10 V	≈4.04	≈185 V	[128]
Vertical SBD	1.6	50.2 mΩ·cm^2^	order 10^−6^–10^−7^ A/cm^2^	≈10 A/cm^2^ @ 10 V	≈10^7^–10^8^	3.3	117 V	[129]
Pseudo-Vertical SBD	2.86–5.17	6.8 mΩ·cm^2^	<10^−6^ A/cm^2^	100–1000 A/cm^2^	NR	NR	1190 V	[130]
Schottky PiN (PiND)	≈4	NR	≈10^−6^ A/cm^2^	>300 A/cm^2^ @ 4 V	>10^9^	NR	>500 V	[131]
PiN	>5	NR	NR	>500 A/cm^2^	∼10^6^–10^7^	>1.25	≈150 V	[132]
High-Voltage PiN	>10	NR	≪μA/cm^2^ at kV	∼10^2^ A/cm^2^	≈10^7^	∼1.9–3.6	up to 11.5 kV	[133]
PND	>5	NR	10^−5^ A/cm^2^	10^−2^ A/cm^2^	10^3^ at ±15 V	NR	NR	[134]
Trenched PND	9	order 10^−2^ mΩ·cm^2^	NR	≈10^6^ A/cm^2^	≈10^8^ @ ±20 V	≈6	≈1370 V	[135]
Vertical PND	5.4	0.415 mΩ·cm^2^	NR	≈10^6^ A/cm^2^	NR	≈6	2.2kV	[136]
PiND	∼4–4.3	0.30 Ω·cm^2^	∼$6\times 10^{-8}\;A/{\mathrm{cm}}^{2}$ @ −10 V	∼$6\;A/{\mathrm{cm}}^{2}$ @ +10 V	∼$10^{8}$ @ ±10	∼3.9	∼207 V	[137]
ITO/diamond n–p heterojunction diode	1.4	13 mΩ·cm^2^	10^−7^–10^−6^ A/cm^2^ at 1 kV	∼100 A/cm^2^	10^11^–10^12^	∼3–4	≈1.1 kV	[138]
Diamond/*ε*-Ga_2_O_3_ heterojunction p–n diode	≈2–3	order 10 mΩ·cm^2^	<1 μA/cm^2^	10^2^–10^3^ A/cm^2^	>10^8^	>6	>3 kV	[139]

**Table 4:** Summary of representative diamond field-effect transistor (FET) architectures reported in the literature. The table covers hydrogen-terminated, oxygen-terminated, deep-depletion, inversion-mode, and junction-controlled devices, highlighting channel formation mechanisms, operation mode, current density, breakdown capability, and key performance trade-offs relevant to high-temperature and high-power applications.

Device Type	Channel/Surface Condition	Operation Mode	Typical On-State Current Density	Breakdown Voltage	Key Feature	Ref.
Hydrogen-terminated MOSFET (H-MOSFET)	Surface 2DHG (C–H termination)	Normally on	Up to ∼1 A/mm	Up to ∼2 kV	High current density, surface instability at elevated temperature	[73]
H-MOSFET with high-*k* dielectric (HfO_2_, ZrO_2_, Al_2_O_3_ stacks)	Surface 2DHG + HfO_2_/ZrO_2_/Al_2_O_3_	Normally on/quasi-off	>100 mA/mm	Up to ∼1–2 kV	Gate-stack engineering to reduce leakage and improve reliability	[76]
Normally off H-MOSFET (Ga_2_O_3_ gate dielectric)	Surface 2DHG + Ga_2_O_3_	Enhancement-mode	>30 mA/mm	NR	Recent progress toward stable normally off operation	[187]
Oxygen-terminated MOSFET (O-MOSFET)	Bulk depletion channel (C–O termination)	Normally off	0.12 μA/mm	1.718 kV	Improved threshold stability, lower current density	[186]
Deep-depletion MOSFET (D^2^MOSFET)	Bulk depletion channel	Normally on or off	1.9μA/mm	∼200 V	Wide depletion region, high-field and high-temperature robustness	[51]
Depletion-controlled fin-structured MOSFET	Fin-based depletion channel	Normally off	Few mA/mm	NR	Electrostatic control and scalability beyond planar MOSFETs	[188]
Inversion-mode MOSFET (boron-doped O-terminated)	Inversion channel, boron-doped body	Normally off	∼1–2 mA/mm	<200 V	Proof-of-concept, high interface trap density	[53]
Vertical diamond MOSFET	Oxidized Si-terminated (C–Si–O) surface and trench-sidewall 2DHG channel	Normally off	108mA/mm	NR	Normally off vertical device using an oxidized Si-terminated diamond channel	[189]
MESFET (Schottky-gated)	Boron-doped bulk channel	Normally on	∼1 mA/mm @ 600 K	>2 kV	Oxide-free gate; high-temperature operation demonstrated	[74]
JFET	p–n junction gate	Normally on/off	Few mA/mm	∼600 V	Junction-controlled channel, with strong reliability potential	[190]
Normally off JFET (submicron channel)	Narrow/lightly doped channel	Normally off	Sub-mA/mm	∼600 V	Enhancement-mode without gate oxide	[191]
2DHG FET (HFET/H-FET)	Surface 2D hole gas	Normally on	>1 A/mm	Limited	Strong RF potential; research-stage devices	[21]

## Data Availability

No new data were created or analyzed in this study. Data sharing is not applicable to this article.
